# Cognitive prehabilitation for older adults undergoing elective surgery: a systematic review and narrative synthesis

**DOI:** 10.3389/fnagi.2024.1474504

**Published:** 2024-10-04

**Authors:** Yu He, Ziliang Wang, Yinuo Zhao, Xiaochai Han, Kangxiang Guo, Nianyi Sun, Xueyong Liu

**Affiliations:** ^1^Department of Rehabilitation, Shengjing Hospital of China Medical University, Shenyang, China; ^2^Department of Physical Medicine and Rehabilitation, The Second Clinical College, China Medical University, Shenyang, China; ^3^Department of Rehabilitation, Shanghai Fourth People’s Hospital, School of Medicine, Tongji University, Shanghai, China; ^4^School of Medicine, Tongji University, Shanghai, China

**Keywords:** aged, elective surgery, prehabilitation, cognition, systematic review

## Abstract

**Background:**

Perioperative cognitive maintenance and protection in older adults is an important patient safety imperative. In addition to foundational care, one area of growing interest is integrating cognitive prehabilitation into the surgical trajectory. This review aimed to evaluate the effectiveness and safety of cognitive prehabilitation on cognitive functional capacity and postoperative cognitive outcomes among older adults undergoing elective surgery.

**Methods:**

The MEDLINE, Embase, CENTRAL, CINAHL, PsycINFO, PEDro, CBM, CNKI, WANFANG, and VIP databases were systematically searched up to September 5, 2024, to identify randomized controlled trials published for English or Chinese. Two authors independently completed the study selection process, data extraction process and methodological quality assessment. The Patient, Intervention, Comparison, Outcome, Study design framework was used to construct the search strategy. The predefined primary outcomes of interest included the incidence of postoperative delirium (POD) and the incidence of delayed neurocognitive recovery (dNCR). The quality of the studies was evaluated by the PEDro scale. Owing to the small number of trials and clinical and methodological diversity, a narrative synthesis was undertaken in accordance with the Synthesis Without Meta-analysis guidelines. This study was conducted and reported in accordance with the Preferred Reporting Items for Systematic Reviews and Meta-analyses statement. The certainty of the evidence was assessed using the Grading of Recommendations Assessment, Development and Evaluation system.

**Results:**

Six studies were analysed. These trials involved 645 total participants, with 316 in the intervention group (mean age, 66.0–73.8 years; 38.4–77.8% male) and 329 in the comparator group (mean age, 67.5–72.6 years; 31.8–88.9% male). The effects of preoperative cognitive training on reducing the incidence of dNCR, the incidence of POD, the length of hospital stay and the incidence of postsurgical complications as well as improving postoperative global cognitive function and activities of daily living are quite uncertain. The results of this study should be interpreted with caution owing to the limited number of trials and low to very low certainty of evidence.

**Conclusion:**

Current evidence on the effectiveness and safety of cognitive prehabilitation on cognitive and noncognitive outcomes in older patients undergoing elective surgery is limited and unclear.

**Systematic review registration:**

https://www.crd.york.ac.uk/prospero/display_record.php?RecordID=277191, Identifier CRD42021277191.

## Introduction

The population of older adults is growing rapidly (United Nations, 2020; Medici, 2021), and the subsequent ever-increasing demand for surgical services among this group presents a specific set of challenges and opportunities for multidisciplinary perioperative health care teams (Lester et al., 2022; Jablonski and Urman, 2019; Berian et al., 2018). Age-related decreases in physiological reserve and functional capacity can increase the risk for surgical complications, postoperative functional decline, and even mortality (Oresanya et al., 2014; Zhang et al., 2020). Anaesthesia and surgery represent “a combined stress” to the brain and may lead to neurocognitive disorders through several perioperative factors, especially in older adults (Chen et al., 2023; Evered et al., 2017; O'Brien et al., 2017). These cognitive impairments were formerly known as postoperative delirium (POD) and postoperative cognitive dysfunction (POCD). More recently, the umbrella term perioperative neurocognitive disorders (PNDs) has been recommended to describe the overall situation, which includes preoperative cognitive impairment, POD occurring in the hours up to 1 week postprocedure or until discharge (whichever occurs first), longer-lasting cognitive decline diagnosed up to 30 days (delayed neurocognitive recovery, dNCR) and up to 12 months after the procedure (postoperative neurocognitive disorder, postoperative NCD) (Evered et al., 2018; Evered et al., 2019). It has been reported that PNDs are associated with an increased risk of multiple adverse outcomes, including increased perioperative and long-term mortality (Evered et al., 2017; Chen et al., 2022; Migirov et al., 2021; Kong et al., 2022), which makes perioperative cognitive maintenance and protection imperative for patient safety (Vacas et al., 2022; Igwe et al., 2020; Peden et al., 2021).

In addition to foundational care (Kehlet, 2008; Ljungqvist et al., 2017; Eamer et al., 2018; Thillainadesan et al., 2022; Fonseca et al., 2021; Chen et al., 2017), one area of growing interest is integrating prehabilitation into the surgical trajectory (Carli et al., 2021; Alvarez-Nebreda et al., 2018; Duro-Ocana et al., 2023; Liu et al., 2022; Norris and Close, 2020). Prehabilitation is the process of augmenting functional reserve prior to scheduled surgery, with the aim of preparing patients to withstand surgical stress and attenuating the functional decline, especially in those with comorbidities and frailty (Wynter-Blyth and Moorthy, 2017; Hulzebos and van Meeteren, 2016; Gillis et al., 2021; López Rodríguez-Arias et al., 2020). While the majority of research has focused on exercise and/or nutrition-based strategies, there is increasing recognition of the need to integrate cognitive support into prehabilitation programs, particularly for the ageing brain (Kong et al., 2022; Hulzebos and van Meeteren, 2016; Rengel et al., 2019; Zietlow et al., 2022; O’Gara et al., 2020). Cognitive prehabilitation, namely preoperative cognitive interventions such as cognitive training, stimulation and rehabilitation, which are aimed at optimising brain function and augmenting cognitive reserve prior to surgery to mitigate PNDs, may have significantly contributed to the perioperative brain function of older patients (O’Gara et al., 2020; Jiang et al., 2024; Rengel et al., 2021; Humeidan et al., 2015). Cognitive interventions are beneficial for improving cognitive function in older adults in various clinical settings (Gavelin et al., 2021; Reijnders et al., 2013; Nguyen et al., 2019; Hill et al., 2017). However, their benefits before surgery within a prehabilitation program are inconclusive (Vlisides et al., 2019; Goettel, 2019).

Although the assessment of cognitive prehabilitation in older elective surgical patients has been gaining increasing attention in recent randomized clinical trials (RCTs), no systematic review has been conducted until now. Therefore, this study aimed to systematically evaluate the current literature on the effectiveness and safety of cognitive prehabilitation programs on cognitive functional capacity and postoperative cognitive outcomes among older adults undergoing elective surgery.

## Methods

### Protocol and registration

We conducted this systematic review according to a previously published protocol (He et al., 2022), that was prospectively registered with the International Prospective Register of Systematic Reviews (PROSPERO) on October 10, 2021, as CRD42021277191. This systematic review was conducted and reported in accordance with the Preferred Reporting Items for Systematic Reviews and Meta-analyses (PRISMA) statement (Page et al., 2021), and the guidelines of the Cochrane Handbook for Systematic Reviews of Interventions (Higgins et al., 2022).

### Inclusion criteria for study selection

As described in our protocol (He et al., 2022), studies were included in the review if they met the following inclusion criteria: (a) Participants–participants aged 60 years and older who were scheduled for elective surgery of any type, with no limits to anaesthetic technique, depth, or agents, regardless of race or sex; (b) Intervention and comparison–cognitive prehabilitation (preoperative cognitive interventions, such as cognitive training, stimulation, and rehabilitation) compared to standard or usual care, or no cognitive prehabilitation under the same perioperative treatment programs; the delivery of preoperative cognitive interventions was not limited to a specific mode (paper-and-pencil, computer-administered, individual, or group-based), and there were no restrictions with respect to setting (hospital, community, or home-based) or intervention dose-related parameters, including the overall duration of the intervention, frequency, and intensity; however, we excluded single-session treatments; (c) Outcome measures—the primary outcomes were the incidence of POD and the incidence of delayed neurocognitive recovery (dNCR); the secondary outcome measures included any validated measure assessing cognitive function, including but not limited to, cognitive screening instruments, such as the Mini-Mental State Examination (MMSE) and Montreal Cognitive Assessment (MoCA), multidomain cognitive assessment scales, neuropsychological test batteries, postsurgical complications, activities of daily living (ADL), health-related quality of life (QOL), length of stay (LOS), mortality (30 days or longer if reported), patient compliance and acceptability, and safety (dropouts, serious adverse events); and (d) Study design and language—RCTs published in English or Chinese. Pilot, multiarm, and cluster RCTs were also eligible. We excluded studies that were research protocols, conference proceedings or abstracts, dissertations, or books as well as studies that lacked available data for analysis.

### Search strategy and study selection

The MEDLINE (via Ovid), Embase (via Ovid), Cochrane Central Register of Controlled Trials (CENTRAL) (via Ovid), Cumulative Index to Nursing and Allied Health Literature (CINAHL) plus (via EBSCOhost), PsycINFO (via EBSCOhost), Physiotherapy Evidence Database (PEDro), Chinese Biomedical Literature Database (CBM), China National Knowledge Infrastructure Library (CNKI), WANFANG Database, and Chinese Scientific Journal Database (VIP) databases were systematically searched from inception to June 10, 2022, and the search was updated on September 5, 2024. The Patient, Intervention, Comparison, Outcome, and Study design (PICOS) framework was used to construct the search strategy: patient (aged), intervention (prehabilitation), outcome (PND) and study design (RCT) (He et al., 2022). Furthermore, we examined the reference lists of the included study reports and any relevant systematic reviews to identify additional eligible studies. The search strategy is shown in Supplementary Table S1.

The retrieved records were imported into EndNote (EndNote Citation Software, Version 9.3, Clarivate Analytics, New York, NY, United States), and duplicate items were removed automatically and manually checked by one author. Two authors independently inspected the titles, abstracts, and keywords of all the articles to eliminate ineligible records. After a preliminary screening, the full texts of the remaining records were retrieved. Two authors independently assessed the full texts in detail according to the predefined criteria and identified studies for inclusion. Discrepancies regarding the eligibility of the selected studies were resolved through discussion or consultation with a third author, if necessary.

### Data extraction and quality assessment

Two authors independently extracted the relevant general information, participant and intervention characteristics, outcome data, and methodological quality components from the included studies using a predetermined data extraction Excel format. The PEDro scale (Maher et al., 2003) has a possible score range of 0–10, with higher scores suggesting higher quality; it was adopted as a criterion to quantify the methodological quality of each included RCT. The scores of each RCT were independently determined by two authors by evaluating the following criteria: randomization, allocation concealment, baseline comparability, blinding, follow-up, intention-to-treat (ITT) analysis, between-group statistical comparisons, and reporting point measures and measures of variability. Discrepancies regarding data extraction and quality assessments were resolved through discussion or consultation with a third author, if necessary.

### Data synthesis

We planned to conduct a quantitative synthesis by means of a meta-analysis. However, given the nature of the extracted data, this was deemed unsuitable to yield a meaningful summary effect estimate owing to the small number of included studies and the clinical and methodological diversity between the trials. Therefore, the available quantitative data were analysed narratively and synthesised in accordance with the Synthesis Without Meta-analysis (SWiM) guidelines (Campbell et al., 2020). The SWiM guidelines include a nine-item checklist designed to promote transparent reporting of systematic reviews that employ narrative synthesis without meta-analysis, which prompts users to report how studies are grouped, any standardised metric used, the synthesis method, how data are presented, a summary of the synthesis findings, and limitations of the synthesis (Campbell et al., 2020). The results of the included studies were summarised and presented through tabulation and description. In addition, we transparently reported, when available, the study outcomes of each trial through structured reporting of effects and calculated effect sizes with 95% confidence intervals (CIs) using RevMan 5 software. We presented the risk ratio (RR) with a 95% CI for dichotomous outcomes and the mean difference (MD) with a 95% CI for continuous outcomes. We applied the Grading of Recommendations Assessment, Development and Evaluation (GRADE) framework to assess the certainty of evidence. The GRADE framework includes five domains: study limitations, indirectness, imprecision, inconsistency and the likelihood of publication bias. Ultimately, the quality of evidence for each outcome falls into one of four levels: high, moderate, low, and very low (Guyatt et al., 2008; Murad et al., 2017). The results are presented in a narrative “Summary of findings” table (Supplementary Table S2).

## Results

### Flow of studies through the review

**Figure 1:**
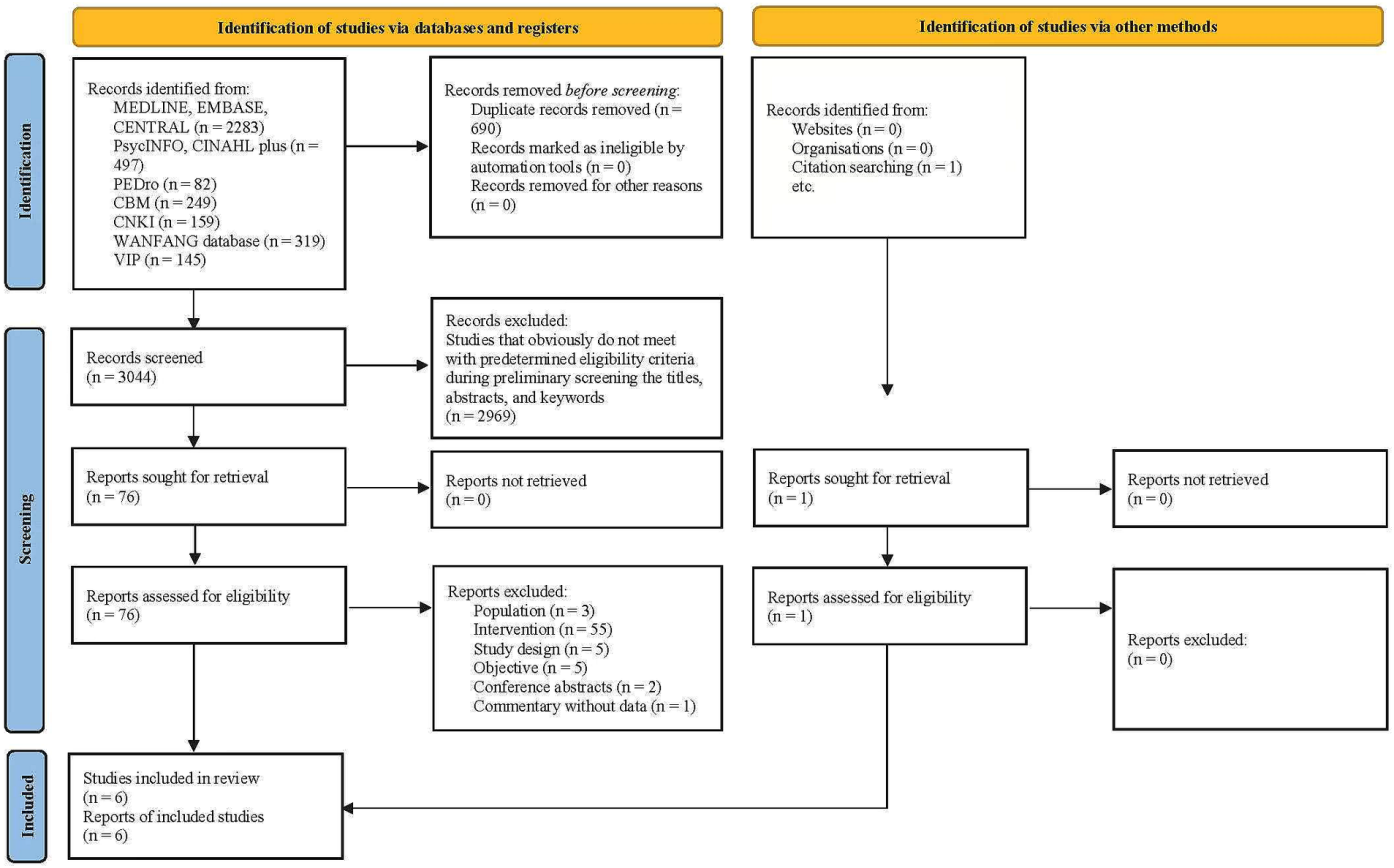
PRISMA flow diagram of the study selection process.

In total, 3,734 records were identified from the database searches, which were reduced to 3,044 after duplicates were removed. After screening titles, abstracts, and keywords, 76 potentially relevant full papers were assessed, with six eligible studies included in this review (Vlisides et al., 2019; Saleh et al., 2015; Humeidan et al., 2021; Li et al., 2018; Wang and Wang, 2017; Greaves et al., 2023) The study selection process is documented in the PRISMA flow diagram (Figure 1). The characteristics of the excluded studies are presented in Supplementary Table S3.

### Characteristics of the included studies

The eligible studies were RCTs that used parallel-arm controls. The studies included 645 older adults, with 316 in the intervention group and 329 in the comparator group. The sample sizes in the included studies ranged from 29 (Greaves et al., 2023) to 251 (Humeidan et al., 2021) participants. The studies were published between 2015 and 2023. Three studies were conducted in China, two in the USA and one in Australia. Of the six included studies, four were published in English and two in Chinese. Further details concerning the characteristics of the included studies are presented in Tables 1 [Table tab2]–3, ordered by study number.

**Table 1:** The main characteristics of participants.

Authors	Country	Funding	Study demographics and characteristics of participants						
			Sample size		Female (%)	Age (Mean/Range)	Baseline education characteristics^c^	Baseline cognition performance^c^	Type of surgery
			*N* ^a^	*N* ^b^					
Vlisides et al. (2019)	USA	The authors have no funding to disclose	61	52	51.92	67	Educational attainment (college or higher): E: 5 (22) C: 12 (41)	Data NR [Older adult with preexisting cognitive impairment (preoperative delirium, mild cognitive impairment, dementia, and/or not having capacity to provide informed consent) was excluded in this study]	Non-cardiac, non-major vascular, non-intracranial surgery: gastrointestinal, urologic, spine, and hepatobiliary surgeries
Saleh et al. (2015)	China	National Natural Scientific Foundation of China (NSFC, 81371216)	147	141	47.52	70.5	Education years: E: 5.7 ± 2.0 C: 6.0 ± 2.4	MMSE (score): E: 28.9 ± 1.5; C: 28.1 ± 1.5 Benton Judgment of Line Orientation Test (score): E: 15.2 ± 1.5; C: 15.3 ± 1.9 Digit Span Test (score): E: 19.1 ± 2.2; C: 19.3 ± 1.7 BVMT-R: E: 10.3 ± 2.1; C: 10.2 ± 1.4 Symbol-Digit Modalities Test (score): E: 24.2 ± 3.6; C: 24.1 ± 3.8 BVMT-R Delayed Recall Test (score): E: 3.4 ± 1.4; C: 3.6 ± 1.2 BVMT-R Recognition Discrimination Index (score): E: 10.3 ± 1.3; C: 10.8 ± 1.2 Trail Making Test (score): E: 193.2 ± 33.1; C: 194.5 ± 35.5 Verbal Fluency Test (score): E: 45.3 ± 6.2; C: 44.7 ± 6.3	Major gastrointestinal surgery
Humeidan et al. (2021)	USA	Institutional funds from the Ohio State University Department of Anesthesiology and Neuroscience Research Institute	268	251	64.94	67.25	Education years: E: 14 (12–16) C: 14 (12–16)	MMSE score: E: 29 (28–30) C: 29 (28–30) Self-Administered Gerocognitive Evaluation score: E: 20 (18–21) C: 19.5 (18–21)	Noncardiac, non-neurological surgery: general, orthopedic, gynecologic, thoracic, urology, plastic, vascular, transplant, and otolaryngology surgeries
Li et al. (2018)	China	Science and technology plan project of colleges and universities of Shandong province (J17KA231); Nursery plan of affiliated hospital of Jining medical university (MP-2014-021)	72	72	44.44	70	Number of illiterate individuals: E: 12 (33.3) C: 15 (41.7)	Data NR (This study adopted MoCA as a measure for preliminary cognitive screening, older adults with MoCA ≥26 was included)	CABG
Wang and Wang (2017)	China	NR	100	100	52	65–75	NR	MMSE (score): E: 28.67 ± 2.02; C: 28.45 ± 2.56	Major gastrointestinal surgery
Greaves et al. (2023)	Australia	National Heart Foundation of Australia Vanguard Grant (101758–VG 2017)	36	29	17	73.2	NR	ACE-III (score): E: 88 ± 8.4; C: 85.7 ± 7	CABG

CABG, coronary artery bypass grafting; MMSE, Mini-Mental State Examination; MoCA, Montreal Cognitive Assessment; BVMT-R, Brief Visuospatial Memory Test-Revised; ACE-III, Addenbrookes Cognitive Examination III; E, experimental group; C, control group; NR, not report.

a Enrolled.

b Analysed.

c Data presented as *n* (%) or mean ± standard deviation or median (interquartile range) unless indicated.

**Table 2 tab2:** The study designs and interventions.

Authors	Setting	Design	Intervention design				Control group
			Program and targeted domains	Dose^a^	Sessions^b^	Duration^c^	
Vlisides et al. (2019)	Home	RCT	An adaptive, home-based, computer-based preoperative cognitive training battery targeting executive function, attention, working memory, and visuospatial processing	2.33	7	20	Usual care
Saleh et al. (2015)	Hospital	RCT	A preoperative cognitive training program with the MoL – a mnemonic technique	3	3	60	Usual care
Humeidan et al. (2021)	Home	RCT	An electronic, tablet-based preoperative cognitive exercise targeting memory, speed, attention, flexibility, and problem-solving	10	10	60	Usual care
Li et al. (2018)	Hospital	RCT	A preoperative cognitive training program targeting memory, orientation, language and attention	NR	3	NR	Usual care
Wang and Wang (2017)	Hospital	RCT	A preoperative cognitive training program targeting orientation, memory, attention, calculation, language and visuospatial function	5	5	60	Usual care
Greaves et al. (2023)	Home	RCT	A computerized cognitive training delivered through the HappyNeuron Pro platform (on a laptop) with selected exercises targeting cognitive domains most affected in heart failure: psychomotor speed, attention, memory and executive function.	2.625–7	3.5–7	45–60	Usual care

RCT, randomized controlled trial; MoL, method of loci; NR, not report.

a Total number of training hours.

b Total number of preoperative cognitive training sessions.

c Session length (minutes).

**Table 3:** The results of individual studies.

Authors	Outcome measure			Key findings^a^	Attrition^b^
	Primary outcome	Secondary outcome	Time to measurement		
Vlisides et al. (2019)	POD incidence (as measured by 3D-CAM)	3 tests from the NIH Toolbox Cognition Battery; LOS; physical therapy session participation	Baseline, preoperative morning, from the PACU through postoperative day 3	PO: POD incidence: E: 6 (26), C: 5 (17), *p* = 0.507 SO: Estimated Mean Score Differences—NIH Toolbox Tests^c^: Flanker Inhibitory Control test: 2.77, 95% CI -1.69 to 7.23, *p* = 0.223; List Sorting Working Memory test: −0.94, 95% CI −5.29 to 3.41, *p* = 0.672; Pattern comparison processing speed: 4.21, 95% CI -3.25 to 11.7, *p* = 0.268 LOS (days): E: 6.8, C: 6.4, *p* = 0.696	E: 23.33% C: 6.45%
Saleh et al. (2015)	dNCR incidence (as measured by a NPT involving 8 cognitive tests)	LOS; postoperative complications	Baseline, 1 week after surgery	PO: dNCR incidence: E: 11 (16), C: 26 (36), *p* = 0.007 SO: LOS (days): E: 12.21 ± 1.90, C: 13.26 ± 1.20, reported no difference was found between groups, no *p* value reported Postoperative complications: neurological: E: 1 (1), C: 2 (3); respiratory: E: 3 (4), C: 2 (3); cardiovascular: E: 2 (3), C: 1 (1); infection: E: 6 (9), C: 8 (11); intensive care unit stay for >24 h: E: 2 (3), C: 3 (4), reported no difference was found between groups, no *p* value reported	E: 6.76% C: 1.37%
Humeidan et al. (2021)	POD incidence (as measured by a brief CAM, MDAS, or a structured medical record review)	LOS Delirium characteristics between groups	Baseline, between postoperative day 0 to day 7 or discharge	PO: POD incidence: E: 18 (14), C: 29 (23), *p* = 0.08 SO: LOS (days): E: 4 (3–6) vs. C: 4 (3–6), *p* = 0.55 Delirium onset: reported no statistical differences in postoperative delirium onset day, *p* = 0.84 delirium duration: E: 2 (1–4), C: 2 (1–4), *p* = 0.91 total delirium-positive days: E: 2 (1–4), C: 2 (1–3), *p* = 0.84	E: 6.72% C: 5.97%
Li et al. (2018)	dNCR incidence (as measured by MoCA)	MoCA score	Baseline, two weeks after intervention	PO: dNCR incidence: E: 6 (17), C: 15 (42), *p* < 0.05 SO: MoCA (score): E: 27.28 ± 1.69, C: 25.76 ± 3.05, *p* < 0.05	E: 0% C: 0%
Wang and Wang (2017)	dNCR incidence (as measured by MMSE)	MMSE score ADL (as measured by BI)	Baseline, before surgery, postoperative day 3 and day 7	PO: dNCR incidence: E: 10 (20), C: 16 (32), *p* < 0.05 (postoperative day 3); E: 6 (12), C: 12 (24), *p* < 0.05 (postoperative day 7) SO: MMSE (score): E: 24.97 ± 1.46, C: 22.13 ± 1.32, *p* < 0.05 (postoperative day 3); E: 26.45 ± 2.77, C: 23.05 ± 1.56, *p* < 0.05 (postoperative day 7) BI: E: 47.39 ± 7.39, C: 41.57 ± 6.96, *p* < 0.05 (postoperative day 3); E: 58.95 ± 6.63, C: 47.82 ± 5.71, *p* < 0.05 (postoperative day 7)	E: 0% C: 0%
Greaves et al. (2023)	POD: CAM-ICU, MDAS	Global cognitive status: ACE-III, CANTAB	Baseline, discharge	Pre-operative CCT did not significantly associate with delirium following CABG surgery (OR = 1.25, 95%CI = [0.30, 5.24], *p* = 0.76); There were no significant effects of CCT on the change in ACE-III (*t* = 0.25, DF = 25.70, *p* = 0.81, *d* = 0.09) and in CANTAB at discharge: executive function (*t* = −0.08, DF = 18.79, *p* = 0.94, *d* = −0.03), psychomotor speed & attention (*t* = 0.43, DF = 22.95, *p* = 0.67, *d* = 0.16), spatial working memory (*t* = −1.76, DF = 21.90, *p* = 0.09, *d* = −0.66).	E: 44% C: 11%

POD, postoperative delirium; dNCR, delayed neurocognitive recovery; NPT, neuropsychological test battery; 3D-CAM, 3 min diagnostic confusion assessment method; CAM, confusion assessment method; CAM-ICU, confusion assessment method for the ICU; MDAS, memorial delirium assessment scale; MMSE, Mini-Mental State Examination; MoCA, Montreal Cognitive Assessment; LOS, length of stay; ADL, activities of daily living; BI, Barthel Index; PO, primary outcome; SO, secondary outcome; PACU, postanesthesia care unit; ACE-III, Addenbrookes Cognitive Examination III; CANTAB, Cambridge Neuropsychological Test Automated Battery; CCT, computerized cognitive training; CABG, coronary artery bypass grafting; CI, confidence interval; E, experimental group; C, control group.

a Data presented as *n* (%) or mean ± standard deviation or median (interquartile range) unless indicated.

b From radomization to post-surgery assessment.

c No group-time interaction terms were significant for any of the tests. Results reflect the mean difference in fully corrected T-scores between groups for each respective test.

#### Participants

The participants’ ages ranged from a mean of 66.0 (Vlisides et al., 2019) to 73.8 years (Greaves et al., 2023) in the intervention group and 67.5 (Humeidan et al., 2021) to 72.6 years (Greaves et al., 2023) in the control group. One study (Wang and Wang, 2017) did not report the mean age of the participants, although it recruited older adults aged between 65.0–75.0 years. The proportion of male participants ranged from 38.4% (Humeidan et al., 2021) to 77.8% (Greaves et al., 2023) in the intervention group and from 31.8% (Humeidan et al., 2021) to 88.9% (Greaves et al., 2023) in the control group.

Four studies reported baseline educational characteristics in terms of years of education (Saleh et al., 2015; Humeidan et al., 2021), educational attainment (college or higher) (Vlisides et al., 2019), and the number of illiterate individuals (Li et al., 2018). All included studies included participants without severe preexisting cognitive impairment. Of these, one study (Li et al., 2018) adopted the MoCA as a measure for preliminary cognitive screening, three studies (Saleh et al., 2015; Humeidan et al., 2021; Wang and Wang, 2017) used the MMSE. The average scores of the MMSE ranged from 28.1 to 29.0 at baseline. In addition, one study (Saleh et al., 2015) reported the baseline scores of eight cognitive tests from a neuropsychological test battery (NPT), and one (Greaves et al., 2023) reported the Addenbrookes Cognitive Examination (ACE-III).

Of the six studies, two studies (Vlisides et al., 2019; Humeidan et al., 2021) recruited participants who were scheduled to undergo elective noncardiac, nonneurological surgery; two studies (Saleh et al., 2015; Wang and Wang, 2017) focused on major gastrointestinal surgery; and the remaining two study (Li et al., 2018; Greaves et al., 2023) included coronary artery bypass grafting surgery. All surgeries were performed under general anaesthesia. Three studies (Saleh et al., 2015; Humeidan et al., 2021; Li et al., 2018) reported the length of surgery, two (Saleh et al., 2015; Li et al., 2018) reported the length of anaesthesia, and Saleh et al. (2015) reported information on estimated blood loss. The main characteristics of the participants are summarised in Table 1.

#### Interventions and comparators

The six included studies delivered individual cognitive training to their intervention participants through trained professionals prior to surgery. The content and structure of the cognitive training programs were diverse. Three studies (Vlisides et al., 2019; Humeidan et al., 2021; Greaves et al., 2023) used preoperative computer-based programs designed to train several cognitive domains in a home-based setting. The other three studies (Saleh et al., 2015; Li et al., 2018; Wang and Wang, 2017) included noncomputer interventions in health care facilities, including cognitive training programs targeting several cognitive domains in two studies (Li et al., 2018; Wang and Wang, 2017) and a mnemonic strategy with the method of loci in one study (Saleh et al., 2015). The control group always received usual or standard perioperative care without cognitive training before surgery.

Overall, the interventions were delivered for at least 3 (Li et al., 2018) to 8 days (Humeidan et al., 2021) preoperatively, mostly at a training frequency of once daily. The total number of training sessions ranged from 3 (Saleh et al., 2015; Li et al., 2018) to 10 (Humeidan et al., 2021), the session duration ranged from 20 (Vlisides et al., 2019) to 60 min (Saleh et al., 2015; Humeidan et al., 2021; Wang and Wang, 2017; Greaves et al., 2023), and the total number of training hours ranged from 2.3 (Vlisides et al., 2019) to 10.0 (Humeidan et al., 2021). The study designs and interventions are summarised in Table 2.

#### Outcome measures

The incidence of delirium from the postanaesthesia care unit (assessment beginning at least 2 h after the end of surgery) through postoperative day 3 (Vlisides et al., 2019), between postoperative day 0 and day 7 or at discharge (Humeidan et al., 2021; Greaves et al., 2023) was reported in three of the included studies. Delirium screening was assessed using the in-person 3 min diagnostic confusion assessment method (3D-CAM) (Vlisides et al., 2019), brief CAM, CAM-ICU, memorial delirium assessment scale (Humeidan et al., 2021; Greaves et al., 2023), or an additional thorough medical record review (Humeidan et al., 2021). Furthermore, in the study by Vlisides et al. (2019), attention, working memory, and processing speed, which are cognitive domains particularly affected by delirium, were assessed perioperatively using three tests from the National Institutes of Health Toolbox Cognition Battery (NIHTB-CB): the Flanker Inhibitory Control and Attention Test, List Sorting Working Memory Test, and the Pattern Comparison Processing Speed Test. Humeidan et al. (2021) compared delirium characteristics between patients in the intervention and control groups.

Three studies evaluated the incidence of dNCR (the term POCD was used in these articles; however, in the present study, we used a new term according to recommended nomenclature) at 1 week after surgery (Saleh et al., 2015), 2 weeks after intervention (Li et al., 2018), or 3 and 7 days after surgery (Wang and Wang, 2017). The three studies adopted different diagnostic methods for dNCR. One study (Saleh et al., 2015) assessed this phenomenon using a one standard deviation decline criterion in an NPT involving eight cognitive tests: the Benton Judgment of Line Orientation Test, Digit Span Test, Brief Visuospatial Memory Test-Revised (BVMT-R), Symbol-Digit Modalities Test, BVMT-R Delayed Recall Test, BVMT-R Recognition Discrimination Index, Trail Making Test (Parts A and B), and Verbal Fluency Test. These tests targeted diverse cognitive domains, such as memory, executive function, attention, processing speed, and language. Patients were defined as having dNCR when they exhibited impairments in two or more of the eight cognitive tests. The remaining two studies used cut-off values of 26 scores for the MoCA (Li et al., 2018), and scores of 24, 20 scores and 17 for educational levels above junior high school, primary school education and illiterate individuals, respectively, for the MMSE (Wang and Wang, 2017), to diagnose dNCR. Greaves et al. (2023) reported cognition outcomes, they assessed addenbrookes cognitive examination III (ACE-III) and cambridge neuropsychological test automated battery (CANTAB) at baseline and discharge.

Regarding other outcomes, three studies reported hospital LOS (Vlisides et al., 2019; Saleh et al., 2015; Humeidan et al., 2021). Saleh et al. (2015) also reported information on postoperative complications. ADL was assessed in only one study (Wang and Wang, 2017). The results and measurement tools of the included studies are summarised in Table 3.

#### Quality

The mean PEDro score of the included studies was 7 (range, 6–8) (Table 4). Overall, these studies were considered to have good methodological quality. All studies had randomly allocated participants, had similar groups at baseline, and reported between-group differences, point estimates and variability. Four studies had fewer than 15% dropouts, Four reported the use of concealed allocation, and three did not report whether an ITT analysis was undertaken. We considered the blinding of participants and intervention providers impossible because the nature of cognitive training-based studies involves training activity before surgery rather than usual perioperative care. Exceptiones are that one study (Saleh et al., 2015), which adopted a preoperative mnemonic strategy, indicated that patients were not informed of the intervention being evaluated, either a neuropsychological assessment or cognitive training; in another study (Greaves et al., 2023), separate consent forms were used for each group (different consent forms for those in intervention vs. control), thus the participants are blinded to the presence of other study groups. Therefore, we awarded one point for this criterion. Five studies had blinded assessors, while the remaining study did not report whether the outcome assessors were blinded. In the study by Humeidan et al. (2021), the anaesthesia care team was mentioned to be blinded, and the others did not comment on whether the anaesthesia care team and surgeons were blinded.

**Table 4:** PEDro criteria and scores for the included trials (*n* = 6).

Author, year	Methodological Quality										Total (0 to 10)
	Randomization	Allocation concealment	Baseline comparability	Participant blinding	Therapist blinding	Assessor blinding	<15% dropouts	ITT analysis	Between-group difference reported	Point estimate and variability reported	
Vlisides et al. (2019)	Y	Y	Y	N	N	Y	N	N	Y	Y	6
Saleh et al. (2015)	Y	Y	Y	Y	N	Y	Y	N	Y	Y	8
Humeidan et al. (2021)	Y	Y	Y	N	N	Y	Y	Y	Y	Y	8
Li et al. (2018)	Y	N	Y	N	N	Y	Y	Y	Y	Y	7
Wang and Wang (2017)	Y	N	Y	N	N	N	Y	Y	Y	Y	6
Greaves et al. (2023)	Y	Y	Y	Y	N	Y	N	N	Y	Y	7

Y, met criteria; N, did not meet criteria; ITT, intention-to-treat analysis.

### Effects of cognitive prehabilitation

#### Incidence of POD

Two studies reported on the incidence of POD (Vlisides et al., 2019; Humeidan et al., 2021). We are uncertain whether cognitive prehabilitation reduces the incidence of POD compared to usual care. Vlisides et al. (2019) did not find any significant differences between the preoperative computer-based cognitive training group and the control group (6/23 [26%] vs. 5/29 [17%], *p* = 0.507) (RR 1.51, 95% CI 0.53 to 4.34; 52 participants; Supplementary Figure S1). Humeidan et al. (2021) reported that tablet-based preoperative cognitive exercise may have little or no effects on the incidence of POD compared to that in the normal daily activity control group (18/125 [14%] vs. 29/126 [23%], *p* = 0.08) (RR 0.63, 95% CI 0.37 to 1.07; 251 participants; Supplementary Figure S1). Greaves et al. (2023) reported incident delirium outcomes that pre-operative CCT did not significantly associate with delirium following CABG surgery (OR = 1.25, 95%CI = [0.30, 5.24], *p* = 0.76). The certainty of the evidence was very low due to concerns about “imprecision”, “inconsistency”, and “publication bias” (Supplementary Table S2).

#### Incidence of dNCR

Three studies evaluated the incidence of dNCR (Saleh et al., 2015; Li et al., 2018; Wang and Wang, 2017). Saleh et al. (2015) reported a significant difference in the incidence of dNCR between the cognitive prehabilitation and control groups (11/69 [16%] vs. 26/72 [36%], *p* = 0.007) (RR 0.44, 95% CI 0.24 to 0.82; 141 participants; Supplementary Figure S2). Li et al. (2018) reported similar results; they compared cognitive prehabilitation with usual perioperative care and found a significantly reduced incidence of dNCR (6/36 [17%] vs. 15/36 [42%], *p* < 0.05) (RR 0.40, 95% CI 0.18 to 0.91; 72 participants; Supplementary Figure S2). In the study by Wang and Wang (2017), cognitive prehabilitation resulted in a lower incidence of dNCR (6/50 [12%] vs. 12/50 [24%]) (RR 0.50, 95% CI 0.20 to 1.23; 100 participants; Supplementary Figure S2). The certainty of the evidence was low due to concerns about “imprecision” and “publication bias” (Supplementary Table S2).

#### Global cognitive function

Global cognitive outcomes were measured using the MMSE (Wang and Wang, 2017) or MoCA (Li et al., 2018) or ACE-III & CANTAB (Greaves et al., 2023) in three studies. Li et al. (2018) reported that cognitive prehabilitation reduced cognitive impairment on the basis of improved MoCA scores 2 weeks after intervention (MD 1.52, 95% CI 0.38 to 2.66; 72 participants; Supplementary Figure S3), and the MD in scores between groups exceeded the minimal clinically important difference (MCID) of 1.22 points (Nasreddine et al., 2005; Wu et al., 2019). Wang and Wang (2017) assessed global cognitive functional performance using the MMSE on postoperative day 7 and reported that MDs significantly differed between groups (MD 3.40, 95% CI 2.52 to 4.28; 100 participants; Supplementary Figure S4), exceeding the MCID of 1.4 points (Howard et al., 2011; Watt et al., 2021). Greaves et al. (2023) found that there were no significant effects of computerized cognitive training on the change in ACE-III (t = 0.25, DF = 25.70, *p* = 0.81, *d* = 0.09) at discharge and in CANTAB at discharge: executive function (t = −0.08, DF = 18.79, *p* = 0.94, *d* = −0.03), psychomotor speed & attention (t = 0.43, DF = 22.95, *p* = 0.67, *d* = 0.16), spatial working memory (t = −1.76, DF = 21.90, *p* = 0.09, *d* = −0.66). The certainty of the evidence was low due to concerns about “imprecision” and “publication bias” (Supplementary Table S2).

#### Other outcomes

The effects of cognitive prehabilitation on noncognitive outcomes (LOS, postoperative complications, ADLs, patient acceptability and compliance, and adverse events) are described in Supplementary Table S4.

## Discussion

### Summary of principal findings

This systematic review identified a small and inconclusive evidence base for the effectiveness and safety of cognitive prehabilitation on postoperative cognitive and noncognitive outcomes in older adults undergoing elective surgery. The effects of preoperative cognitive training on reducing the incidence of dNCR, the incidence of POD and LOS, the incidence of postsurgical complications, as well as improving postoperative global cognitive function and ADLs are quite uncertain. The results of this systematic review should be interpreted with caution because of the limited number of trials and the low to very low certainty of evidence.

### Issues related to the definition and scope of interventions

Cognitive interventions are diverse nonpharmacological therapies based upon the distinct theoretical constructs aimed at preventing decline, restoring reduced function, and compensating for impairment (Sikkes et al., 2021). In our study, all six included studies investigated the effect of cognitive training, which typically refers to guided practice on a set of standardised tasks targeting a specific cognitive domain or domain such as memory, attention, or problem solving, and is intended to benefit cognitive functions (Clare et al., 2003; Bahar-Fuchs et al., 2013). It falls within the scope of complex intervention and needs to be developed and evaluated under the guidance of frameworks such as the Medical Research Council complex intervention research framework (Craig et al., 2008; Skivington et al., 2021). While it has often been the focus of research with clinical populations, such as those with dementia and mild cognitive impairment, there is increasing evidence about its utility in preventing cognitive decline (Butler et al., 2018; Gates et al., 2019), with the possible mechanism built on the premise of cognitive reserve (Stern, 2012).

In our study, the content and structure of the preoperative cognitive training were clinically heterogeneous, with most targeting several cognitive domains simultaneously and the other one single cognitive domain, some delivered through paper-and-pencil and others via computer-based platforms. The settings at which the interventions were delivered were also diverse, with some delivered at supervised hospital settings and others at home without supervision. Notably, the actual total training time was predominantly less than 5 h, which falls short of the 10 h presumed to be the effective “dose” of cognitive training (Willis et al., 2006; Edwards et al., 2018). Due to the limited studies included, core/specific ingredients and dosing parameters, especially the minimal effective dosage prior to surgery and the most effective and/or feasible type of cognitive interventions, remain to be investigated in future studies.

### Participant adherence

Attrition is frequently a barrier to cognitive training trials, particularly in unsupervised, home-based and computer-based settings. Therefore, the interpretation of the actual effects of the interventions may be biased in the context of suboptimal compliance. Although expecting perfect adherence may not be realistic, there is a need for supervision or intervention of home-based training by utilising devices with cellular capabilities that may allow for real-time analysis, automated reminders, targeted coaching, and more customised training in future studies (O’Gara et al., 2020; Humeidan et al., 2021).

Although cognitive prehabilitation offers hope for preemptive neurocognitive optimisation, it also raises awareness of how challenging it may be to integrate it in the surgical trajectory. This is also true for its physical counterpart, the compliance rates of which vary from 16 to 97% (Bruns et al., 2016; Punnoose et al., 2023; Carli et al., 2020). The identification of support and barriers to adherence is an important step towards the development and implementation of prehabilitation (Ng et al., 2022). Surveys of surgical patients have shown that factors related to surgical prehabilitation program adherence include patient-centered programs with tailored interventions (Wynter-Blyth and Moorthy, 2017; Ferreira et al., 2018; Gurunathan et al., 2023), interpersonal and environmental motivators (Wynter-Blyth and Moorthy, 2017; Parker et al., 2019; Gillis et al., 2021; Cooper et al., 2022), patient empowerment, and understanding the importance of prehabilitation (Cooper et al., 2022; Porter Starr et al., 2023). In terms of cognitive components, investigations targeting attitudes and perceptions towards cognitive training in older adults indicated that support strategies (e.g., regular personalized feedback and supervision, assistant service) (Trenorden et al., 2022; Beishon et al., 2022; Chen et al., 2021), identification and management of lack of motivation, low mood, and anxiety and depression (Beishon et al., 2022), as well as incorporating meaningful social engagement (Stephan et al., 2024), contributed to positive participant experiences. Therefore, protocol adherence may be a key mediator of prehabilitation efficacy; it is crucial to understand and address this barrier in future research.

### Measures and outcomes

Notably, the diagnostic tools and rules for delirium and dNCR differed across the included studies, which may partially explain their heterogeneity. This was consistent with the findings of the study by Borchers et al. (2021). This finding again confirms the importance of using a more scientific approach to define the outcomes measured in clinical research. Future efforts to define standards for perioperative cognition research, including diagnostic criteria or rules as well as core outcome sets, especially those better aligned with the recent American Society of Anesthesiologists statement on Perioperative Brain Health and recommendations of the Nomenclature Consensus Working Group, are warranted (Evered et al., 2018; Evered et al., 2019; Borchers et al., 2021; Gargon, 2016). The appropriate clinical interpretation of changes on an outcome must consider not only statistical significance, but also whether the observed change is of genuine clinical value to patients (McGlothlin and Lewis, 2014; Embry and Piccirillo, 2020). MCID defines the smallest amount an outcome must change to be meaningful to patients that demonstrates a clinical benefit of an intervention (McGlothlin and Lewis, 2014; Harris et al., 2023). The MCID can be determined using consensus, anchor, and distribution-based methods (McGlothlin and Lewis, 2014). It should also be noted that very few of the included studies defined or mentioned an MCID, or reported data on psychological well-being, QOL, daily functioning, and adverse events, which are important outcomes in clinical decision-making.

### Agreements and disagreements with other studies or reviews

The results of our study are similar to those of four recent reviews in related fields (Daksla et al., 2022; Volz et al., 2022; Jiang et al., 2023; Zhao et al., 2024; Bowden et al., 2023), although these reviews reported on heterogeneous populations (Daksla et al., 2022; Volz et al., 2022; Jiang et al., 2023; Zhao et al., 2024; Bowden et al., 2023), or with indications for oncological or gynaecological surgery (Daksla et al., 2022; Volz et al., 2022), or targeting on postoperative cognitive interventions (Jiang et al., 2023; Bowden et al., 2023). The findings revealed that preoperative cognitive training might be a useful addition to multimodal surgical prehabilitation in perioperative pathways. Comments for two of the included trials (Vlisides et al., 2019; Humeidan et al., 2021) also reflected that cognitive prehabilitation could be beneficial for older surgical patients (Goettel, 2019; Keshock, 2022). However, this question remains to be answered given the paucity of data and the limitations that prevent researchers from drawing firm conclusions. With increasing awareness of the importance of optimising perioperative brain health, the importance of cognitive prehabilitation for the growing surgical population of older adult needs to be further demonstrated by future studies (Culley and Crosby, 2015; Lenze et al., 2019; Kato and Solt, 2021).

### Limitations

This study has some limitations that hinder the interpretability of the results. First, the limited number of studies and the heterogeneity among them precluded us from performing a planned quantitative synthesis. Second, despite conducting systematic searches in multiple relevant databases, our searches were restricted to studies published in the English and Chinese (the authors’ native language), and we did not search for grey literature, as prior studies have shown that excluding unpublished studies, dissertations (Schmucker et al., 2017; Hartling et al., 2017), and non-English (Hartling et al., 2017; Dobrescu et al., 2021; Nussbaumer-Streit et al., 2020) publications would have little impact on the overall conclusions. However, this may have resulted in relevant studies being missed, and the resulting language and publication bias may compromise the comprehensiveness and timeliness of this study. Moreover, there were minor amendments to the published review protocol, and we did not report any meta-analyses due to clinical and methodological heterogeneity, which may have led to misleading results that lacked generalizability. The differences between the protocols and reviews are described in Supplementary Table S5.

### Implications for future research

First, fundamental and early-stage efforts are needed to develop or identify cognitive prehabilitation regimens with greater acceptance of the participants, followed by preliminary trials with the aim of feasibility testing, evaluation, and implementation of intervention strategies. Important questions should focus on setting, timing, type, duration, frequency, and intensity as well as the minimum effective dosage needed to exert clinically important benefits. These findings will serve as a foundation for future well-designed, executed, and reported large-scale, appropriately powered RCTs in this field (Lobo et al., 2023). In addition, future studies should address the issues raised in the present review. At present, cognitive prehabilitation studies mainly involve noncardiac, nonneurological surgery, and more information is needed for other surgical specialties. Preoperative patient characteristics, including cognitive status, education, comorbidities, and medications, can provide valuable information for outcome and risk stratification. Studies should also report compliance with intervention strategies so that outcomes can be pooled or stratified according to the degree of compliance. Future studies should be better powered to demonstrate the cost effectiveness of cognitive prehabilitation. It is also important to standardise validated cognitive outcome measures in future research to decrease between-study heterogeneity and enable pooled analysis. This is further highlighted by the importance of initiating core outcome measures for perioperative and anaesthetic care (Boney et al., 2022) and mapping these alongside standardised endpoints in perioperative medicine (Moonesinghe et al., 2019; Haller et al., 2019). Finally, trials with prolonged follow-up are required to understand both the short- and longer-term beneficial effects of cognitive prehabilitation.

## Conclusion

In conclusion, current evidence of the effectiveness of cognitive prehabilitation on cognitive and noncognitive outcomes in older patients undergoing elective surgery is limited and uncertain. Future studies should be encouraged to address current limitations, as well as issues raised in the present review, to provide a more certain understanding of the effects of cognitive prehabilitation on older adults preparing for surgery. Important aims should include determining which groups of people (according to risk stratification) and types of surgery benefit the most and investigating the type, setting, timing of preoperative cognitive interventions, minimum number of sessions and duration needed to exert clinically important benefits. It is also important to understand intervention adherence, postoperative complications, QOL, adverse effects, prolonged effectiveness, and cost implications. Further research is necessary to determine a consensus on core outcome measures and the MCIDs for each.

## Data Availability

The original contributions presented in the study are included in the article/Supplementary material, further inquiries can be directed to the corresponding authors.
