# Supplementary material for: Cognitive prehabilitation for older adults undergoing elective surgery: a systematic review and narrative synthesis
Source: Front Aging Neurosci. 2024 Oct 4;16:1474504. doi: 10.3389/fnagi.2024.1474504 (PMC11486734; doi:10.3389/fnagi.2024.1474504)
Supplement: Supplementary file 1 [file Data_Sheet_1.docx]

**Supplementary materials**

**Table 1.** Search strategy

**Table 2.** Summary of findings.

**Table 3.** Characteristics of excluded studies.

**Figure 1.** Cognitive prehabilitation effects on the incidence of POD.

**Figure 2.** Cognitive prehabilitation effects on the incidence of dNCR.

**Figure 3.** Global cognitive function: MoCA.

**Figure 4.** Global cognitive function: MMSE.

**Table 4.** Effects of cognitive prehabilitation: other outcomes

**Figure 5.** Length of hospital stay.

**Figure 6.** Incidence of neurological complications.

**Figure 7.** Incidence of respiratory complications.

**Figure 8.** Incidence of cardiovascular complications.

**Figure 9.** Incidence of infection.

**Figure 10.** Incidence of intensive care unit stay for >24 h.

**Figure 11.** ADL immediately before surgery.

**Figure 12.** ADL at 7 days after surgery.

**Table 5.** Differences between protocol and review.

**Table 1.** Search strategy

Search strategy (Initial search on June 10, 2022)

| **A. MEDLINE, CENTRAL, and EMBASE (via Ovid) search**  Database: Embase <1974 to 2022 June 10>, MEDLINE(R) All including Epub Ahead of Print, In-Process & Other Non-Indexed Citations, Daily and Versions(R) <1946-current>, EBM Reviews - Cochrane Central Register of Controlled Trials <May 2022>  Search Strategy:  1 exp perioperative period/ (171666)  2 exp perioperative care/ (231434)  3 (Pre-operati* or Preoperati* or Pre operati* or Pre-surg* or Presurg* or Pre surg* or prior to surgery or before surgery or Pre-operative Rehabilitat* or Preoperative Rehabilitat* or Pre operative Rehabilitat* or Pre-operative Conditioning* or Preoperative Conditioning* or Pre operative Conditioning* or Pre-operative training or Preoperative training or Pre operative training or Pre-operative Exercise* or Preoperative Exercise* or Pre operative Exercise* or Prehabilitat* or Pre-habilitat* or Pre habilitat* or Prophylactic rehabilitat* or Preventive rehabilitat*).ab,ti. (1067597)  4 1 or 2 or 3 (1308191)  5 exp postoperative cognitive complications/ (2040)  6 exp delirium/ (49762)  7 (postoperative cognitive complication* or postoperative cognitive dysfunction* or postoperative decline* or post-surgical cognitive complication* or post-surgical cognitive dysfunction* or postoperative delirium or post-surgical delirium or acute confus* or acute organic psychosyndrome* or metabolic encephalopath* or acute psycho-organic syndrome* or clouded state* or clouding of consciousness or exogenous psycho* or acute brain dysfunction or toxic psycho* or toxic confusion* or toxic encephalopath* or exogenous psycho* or perioperative neurocognitive disorder* or postoperative neurocognitive disorder* or post-surgical neurocognitive disorder* or PND or POCD or POD).ab,ti. (62202)  8 5 or 6 or 7 (106516)  9 exp randomized controlled trial/ (1285298)  10 randomized controlled trial.pt. (1128690)  11 controlled clinical trial.pt. (188100)  12 (random* or placebo or sham or trial).ab,ti. (5515426)  13 9 or 10 or 11 or 12 (5902822)  14 exp aged/ (7004474)  15 exp "aged, 80 and over"/ (1325531)  16 (elderly or senior or seniors or older adult or older adults or geriatric or geriatrics or old people or older-age or old age or adult or adults or older people).ab,ti. (4354566)  17 14 or 15 or 16 (10144206)  18 4 and 8 and 13 and 17 (1978)  19 remove duplicates from 18 (1118) |
| --- |
| **B. PsycINFO, and CINAHL plus (via EBSCOhost) search**  Database: CINAHL plus with Full Text; PsycINFO  1 SU perioperative period OR SU (perioperative care or perioperative nursing ) OR AB ( Pre-operati* OR Preoperati* OR Pre operati* OR Presurg* OR Presurg* OR Pre surg* OR prior to surgery OR before surgery OR Pre-operative Rehabilitat* OR Preoperative Rehabilitat* OR Preoperative Rehabilitat* OR Preoperative Conditioning* OR Preoperative Conditioning* OR Preoperative Conditioning* OR Preoperative training OR Preoperative training OR Pre operative training OR Pre-operative Exercise* OR Preoperative Exercise* OR Preoperative Exercise* OR Prehabilitat* OR Pre-habilitat* OR Pre habilitat* OR Prophylactic rehabilitat* OR Preventive rehabilitat* ) (Search Options: Limiters - Randomized Controlled Trials; Expanders - Apply equivalent subjects; Search modes – Boolean/Phrase) (16535)  2 SU postoperative cognitive complications OR SU ( delirium or acute confusion or confusion or disorientation ) OR AB ( postoperative cognitive complication* OR postoperative cognitive dysfunction* OR postoperative decline* OR postsurgical cognitive complication* OR post-surgical cognitive dysfunction* OR postoperative delirium OR postsurgical delirium OR acute confus* OR acute organic psychosyndrome* OR metabolic encephalopath* OR acute psycho-organic syndrome* OR clouded state* OR clouding of ﻿consciousness OR exogenous psycho* OR acute brain dysfunction OR toxic psycho* OR toxic confusion* OR toxic encephalopath* OR exogenous psycho* OR perioperative neurocognitive disorder* OR postoperative neurocognitive disorder* OR post-surgical neurocognitive disorder* OR PND OR POCD OR POD ) (Search Options: Limiters - Randomized Controlled Trials; Expanders - Apply equivalent subjects; Search modes – Boolean/Phrase) (12642)  3 SU aged OR SU (aged, 80 and over ) OR AB ( elderly OR senior OR seniors OR older adult OR older adults OR geriatric OR geriatrics OR old people OR older-age OR old age OR adult OR adults OR older people ) (Search Options: Limiters - Randomized Controlled Trials; Expanders - Apply equivalent subjects; Search modes – Boolean/Phrase) (905103)  4 1 AND 2 AND 3 (422) |
| **C. PEDro database**  Abstract & Title (prehabilitation or preoperative) AND Subdiscipline (gerontology) AND Method (clinical trial) (76) |
| **D. CBM, CNKI, WANFANG database and VIP search**  1. Terms specific to prehabilitation  篇名,关键词,摘要(预处理) OR篇名,关键词,摘要(预康复) OR篇名,关键词,摘要(围术期康复)  2. Terms specific to surgery  篇名,关键词,摘要(手术)  3. Terms specific to aged  篇名,关键词,摘要(老年)  4. Terms specific to cognition  篇名,关键词,摘要(认知)  5. 1 AND 2 AND 3 AND 4 (CBM 129+CNKI 134+VIP 85+WANGFANG 229=577) |

Search strategy (Updated search on September 6, 2023).

| **A. MEDLINE, CENTRAL, and EMBASE (via Ovid) search**  Database: Embase <1974 to 2023 September 06>, Ovid MEDLINE(R) ALL <1946 to September 06, 2023>, EBM Reviews - Cochrane Central Register of Controlled Trials <August 2023>  Search Strategy:  1 exp perioperative period/ (1267041)  2 limit 1 to yr="2022 -Current" (132545)  3 exp perioperative care/ (330278)  4 limit 3 to yr="2022 -Current" (7371)  5 (Pre-operati* or Preoperati* or Pre operati* or Pre-surg* or Presurg* or Pre surg* or prior to surgery or before surgery or Pre-operative Rehabilitat* or Preoperative Rehabilitat* or Pre operative Rehabilitat* or Pre-operative Conditioning* or Preoperative Conditioning* or Pre operative Conditioning* or Pre-operative training or Preoperative training or Pre operative training or Pre-operative Exercise* or Preoperative Exercise* or Pre operative Exercise* or Prehabilitat* or Pre-habilitat* or Pre habilitat* or Prophylactic rehabilitat* or Preventive rehabilitat*).ab,ti. (1161075)  6 limit 5 to yr="2022 -Current" (128673)  7 2 or 4 or 6 (220885)  8 exp postoperative cognitive complications/ (2511)  9 limit 8 to yr="2022 -Current" (600)  10 exp delirium/ (55942)  11 limit 10 to yr="2022 -Current" (7519)  12 (postoperative cognitive complication* or postoperative cognitive dysfunction* or postoperative decline* or post-surgical cognitive complication* or post-surgical cognitive dysfunction* or postoperative delirium or post-surgical delirium or acute confus* or acute organic psychosyndrome* or metabolic encephalopath* or acute psycho-organic syndrome* or clouded state* or clouding of consciousness or exogenous psycho* or acute brain dysfunction or toxic psycho* or toxic confusion* or toxic encephalopath* or exogenous psycho* or perioperative neurocognitive disorder* or postoperative neurocognitive disorder* or post-surgical neurocognitive disorder* or PND or POCD or POD).ab,ti. (70705)  13 limit 12 to yr="2022 -Current" (11171)  14 9 or 11 or 13 (17311)  15 exp randomized controlled trial/ (1410187)  16 limit 15 to yr="2022 -Current" (137391)  17 randomized controlled trial.pt. (599327)  18 limit 17 to yr="2022 -Current" (40526)  19 controlled clinical trial.pt. (95417)  20 limit 19 to yr="2022 -Current" (722)  21 (random* or placebo or sham or trial).ab,ti. (5981975)  22 limit 21 to yr="2022 -Current" (660694)  23 16 or 18 or 20 or 22 (664943)  24 exp aged/ 7354929  25 limit 24 to yr="2022 -Current" 410360  26 exp "aged, 80 and over"/ (1370360)  27 limit 26 to yr="2022 -Current" (55738)  28 (elderly or senior or seniors or older adult or older adults or geriatric or geriatrics or old people or older-age or old age or adult or adults or older people).ab,ti. (4735603)  29 limit 28 to yr="2022 -Current" (529453)  30 25 or 27 or 29 (808038)  31 7 and 14 and 23 and 30 (576)  32 remove duplicates from 31 (427)  **B. PsycINFO, and CINAHL plus (via EBSCOhost) search**  Database: CINAHL plus with Full Text; PsycINFO  1 SU perioperative period OR SU (perioperative care or perioperative nursing ) OR AB ( Pre-operati* OR Preoperati* OR Pre operati* OR Presurg* OR Presurg* OR Pre surg* OR prior to surgery OR before surgery OR Pre-operative Rehabilitat* OR Preoperative Rehabilitat* OR Preoperative Rehabilitat* OR Preoperative Conditioning* OR Preoperative Conditioning* OR Preoperative Conditioning* OR Preoperative training OR Preoperative training OR Pre operative training OR Pre-operative Exercise* OR Preoperative Exercise* OR Preoperative Exercise* OR Prehabilitat* OR Pre-habilitat* OR Pre habilitat* OR Prophylactic rehabilitat* OR Preventive rehabilitat* ) (Search Options: Limiters - Published Date: 20220601-20241231; Publication Year: 2022-2024; Publication Type: Randomized Controlled Trials; Expanders - Apply equivalent subjects; Search modes – Boolean/Phrase) (900)  2 SU postoperative cognitive complications OR SU ( delirium or acute confusion or confusion or disorientation ) OR AB ( postoperative cognitive complication* OR postoperative cognitive dysfunction* OR postoperative decline* OR postsurgical cognitive complication* OR post-surgical cognitive dysfunction* OR postoperative delirium OR postsurgical delirium OR acute confus* OR acute organic psychosyndrome* OR metabolic encephalopath* OR acute psycho-organic syndrome* OR clouded state* OR clouding of consciousness OR exogenous psycho* OR acute brain dysfunction OR toxic psycho* OR toxic confusion* OR toxic encephalopath* OR exogenous psycho* OR perioperative neurocognitive disorder* OR postoperative neurocognitive disorder* OR post-surgical neurocognitive disorder* OR PND OR POCD OR POD ) (Search Options: Limiters - Published Date: 20220601-20241231; Publication Year: 2022-2024; Publication Type: Randomized Controlled Trials; Expanders - Apply equivalent subjects; Search modes – Boolean/Phrase) (555)  3 SU aged OR SU (aged, 80 and over ) OR AB ( elderly OR senior OR seniors OR older adult OR older adults OR geriatric OR geriatrics OR old people OR older-age OR old age OR adult OR adults OR older people ) (Search Options: Limiters - Published Date: 20220601-20241231; Publication Year: 2022-2024; Publication Type: Randomized Controlled Trials; Expanders - Apply equivalent subjects; Search modes – Boolean/Phrase) (26911)  4 1 AND 2 AND 3 (39) |
| --- |
| **C. PEDro database**  Abstract & Title (prehabilitation or preoperative) AND Subdiscipline (gerontology) AND Method (clinical trial) AND Published Since 2022 (6) |
| **D. CBM, CNKI, WANFANG database and VIP search**  1. Terms specific to prehabilitation  篇名,关键词,摘要(预处理) OR篇名,关键词,摘要(预康复) OR篇名,关键词,摘要(围术期康复)  2. Terms specific to surgery  篇名,关键词,摘要(手术)  3. Terms specific to aged  篇名,关键词,摘要(老年)  4. Terms specific to cognition  篇名,关键词,摘要(认知)  5. Published Year  时间范围(2022-至今)  6. 1 AND 2 AND 3 AND 4 AND 5 (CBM 40+CNKI 13+VIP 28+WANGFANG 61=142) |

Search strategy (Updated search on September 5, 2024).

| **1. MEDLINE (via Ovid) search**  Ovid MEDLINE(R) ALL <1946 to September 03, 2024>  1 exp perioperative period/ 104876  2 exp perioperative care/ 160956  3 (Pre-operati* or Preoperati* or Pre operati* or Pre-surg* or Presurg* or Pre surg* or prior to surgery or before surgery or Pre-operative Rehabilitat* or Preoperative Rehabilitat* or Pre operative Rehabilitat* or Pre-operative Conditioning* or Preoperative Conditioning* or Pre operative Conditioning* or Pre-operative training or Preoperative training or Pre operative training or Pre-operative Exercise* or Preoperative Exercise* or Pre operative Exercise* or Prehabilitat* or Pre-habilitat* or Pre habilitat* or Prophylactic rehabilitat* or Preventive rehabilitat*).ab,ti. 482676  4 1 or 2 or 3 666399  5 exp postoperative cognitive complications/ 614  6 exp delirium/ 13650  7 (postoperative cognitive complication* or postoperative cognitive dysfunction* or postoperative decline* or post-surgical cognitive complication* or post-surgical cognitive dysfunction* or postoperative delirium or post-surgical delirium or acute confus* or acute organic psychosyndrome* or metabolic encephalopath* or acute psycho-organic syndrome* or clouded state* or clouding of consciousness or exogenous psycho* or acute brain dysfunction or toxic psycho* or toxic confusion* or toxic encephalopath* or exogenous psycho* or perioperative neurocognitive disorder* or postoperative neurocognitive disorder* or post-surgical neurocognitive disorder* or PND or POCD or POD).ab,ti. 30895  8 5 or 6 or 7 42070  9 exp randomized controlled trial/ 622463  10 randomized controlled trial.pt. 620839  11 controlled clinical trial.pt. 95599  12 (random* or placebo or sham or trial).ab,ti. 2060686  13 9 or 10 or 11 or 12 2215614  14 exp aged/ 3561946  15 (elderly or senior or seniors or older adult or older adults or geriatric or geriatrics or old people or older-age or old age or adult or adults or older people).ab,ti. 2038319  16 14 or 15 5011031  17 4 and 8 and 16 3062  18 limit 17 to yr="2023 -Current" 390  **2. CENTRAL (via Cochrane Liabrary) search**  Cochrane Central Register of Controlled Trials <Issue 8 of 12, August 2024>  #1 MeSH descriptor: [Perioperative Period] explode all trees 11536  #2 MeSH descriptor: [Perioperative Care] explode all trees 15236  #3 (Pre-operati* or Preoperati* or Pre operati* or Pre-surg* or Presurg* or Pre surg* or prior to surgery or before surgery or Pre-operative Rehabilitat* or Preoperative Rehabilitat* or Pre operative Rehabilitat* or Pre-operative Conditioning* or Preoperative Conditioning* or Pre operative Conditioning* or Pre-operative training or Preoperative training or Pre operative training or Pre-operative Exercise* or Preoperative Exercise* or Pre operative Exercise* or Prehabilitat* or Pre-habilitat* or Pre habilitat* or Prophylactic rehabilitat* or Preventive rehabilitat*):ti,ab,kw in Trials (Word variations have been searched) 141714  #4 #1 or #2 or #3 154542  #5 MeSH descriptor: [Postoperative Cognitive Complications] explode all trees 131  #6 MeSH descriptor: [Delirium] explode all trees 1634  #7 (postoperative cognitive complication* or postoperative cognitive dysfunction* or postoperative decline* or post-surgical cognitive complication* or post-surgical cognitive dysfunction* or postoperative delirium or post-surgical delirium or acute confus* or acute organic psychosyndrome* or metabolic encephalopath* or acute psycho-organic syndrome* or clouded state* or clouding of consciousness or exogenous psycho* or acute brain dysfunction or toxic psycho* or toxic confusion* or toxic encephalopath* or exogenous psycho* or perioperative neurocognitive disorder* or postoperative neurocognitive disorder* or post-surgical neurocognitive disorder* or PND or POCD or POD):ti,ab,kw in Trials (Word variations have been searched) 13278  #8 #5 or #6 or #7 13936  #9 MeSH descriptor: [Aged] explode all trees 278605  #10 (elderly or senior or seniors or older adult or older adults or geriatric or geriatrics or old people or older-age or old age or adult or adults or older people):ti,ab,kw in Trials (Word variations have been searched) 968831  #11 #9 or #10 1038172  #12 MeSH descriptor: [Randomized Controlled Trial] explode all trees 37  #13 (random* or placebo or sham or trial):ti,ab,kw in Trials (Word variations have been searched) 1599350  #14 #12 or #13 1599350  #15 #4 and #8 and #11 and #14 with Publication Year from 2023 to 2024, with Cochrane Library publication date Between Sep 2023 and Sep 2024, in Trials 251  **3. EMBASE (via Ovid) search**  Embase <1988 to 2024 Week 35>  1 exp perioperative period/ 1191111  2 exp perioperative care/ 156006  3 (Pre-operati* or Preoperati* or Pre operati* or Pre-surg* or Presurg* or Pre surg* or prior to surgery or before surgery or Pre-operative Rehabilitat* or Preoperative Rehabilitat* or Pre operative Rehabilitat* or Pre-operative Conditioning* or Preoperative Conditioning* or Pre operative Conditioning* or Pre-operative training or Preoperative training or Pre operative training or Pre-operative Exercise* or Preoperative Exercise* or Pre operative Exercise* or Prehabilitat* or Pre-habilitat* or Pre habilitat* or Prophylactic rehabilitat* or Preventive rehabilitat*).ab,ti. 657963  4 1 or 2 or 3 1540567  5 exp postoperative cognitive complications/ 2268  6 exp delirium/ 43872  7 (postoperative cognitive complication* or postoperative cognitive dysfunction* or postoperative decline* or post-surgical cognitive complication* or post-surgical cognitive dysfunction* or postoperative delirium or post-surgical delirium or acute confus* or acute organic psychosyndrome* or metabolic encephalopath* or acute psycho-organic syndrome* or clouded state* or clouding of consciousness or exogenous psycho* or acute brain dysfunction or toxic psycho* or toxic confusion* or toxic encephalopath* or exogenous psycho* or perioperative neurocognitive disorder* or postoperative neurocognitive disorder* or post-surgical neurocognitive disorder* or PND or POCD or POD).ab,ti. 41753  8 5 or 6 or 7 81215  9 exp randomized controlled trial/ 826087  10 randomized controlled trial.pt. 0  11 controlled clinical trial.pt. 0  12 (random* or placebo or sham or trial).ab,ti. 2762160  13 9 or 10 or 11 or 12 2852767  14 exp aged/ 3764718  15 (elderly or senior or seniors or older adult or older adults or geriatric or geriatrics or old people or older-age or old age or adult or adults or older people).ab,ti. 2599618  16 14 or 15 5612085  17 4 and 8 and 16 9274  18 limit 17 to ("remove medline records" and (clinical trial or randomized controlled trial or controlled clinical trial) and yr="2023 - 2024") 97  **4. PsycINFO (via EBSCOhost) search**  Database: PsycINFO  1 SU perioperative period OR SU (perioperative care or perioperative nursing ) OR AB ( Pre-operati* OR Preoperati* OR Pre operati* OR Presurg* OR Presurg* OR Pre surg* OR prior to surgery OR before surgery OR Pre-operative Rehabilitat* OR Preoperative Rehabilitat* OR Preoperative Rehabilitat* OR Preoperative Conditioning* OR Preoperative Conditioning* OR Preoperative Conditioning* OR Preoperative training OR Preoperative training OR Pre operative training OR Pre-operative Exercise* OR Preoperative Exercise* OR Preoperative Exercise* OR Prehabilitat* OR Pre-habilitat* OR Pre habilitat* OR Prophylactic rehabilitat* OR Preventive rehabilitat* ) (Search Options: Limiters - Publication Date: 20230901- 20240931; Expanders - Apply equivalent subjects; Search modes – Proximity) (384)  2 SU postoperative cognitive complications OR SU ( delirium or acute confusion or confusion or disorientation ) OR AB ( postoperative cognitive complication* OR postoperative cognitive dysfunction* OR postoperative decline* OR postsurgical cognitive complication* OR post-surgical cognitive dysfunction* OR postoperative delirium OR postsurgical delirium OR acute confus* OR acute organic psychosyndrome* OR metabolic encephalopath* OR acute psycho-organic syndrome* OR clouded state* OR clouding of consciousness OR exogenous psycho* OR acute brain dysfunction OR toxic psycho* OR toxic confusion* OR toxic encephalopath* OR exogenous psycho* OR perioperative neurocognitive disorder* OR postoperative neurocognitive disorder* OR post-surgical neurocognitive disorder* OR PND OR POCD OR POD ) (Search Options: Limiters - Publication Date: 20230901- 20240931; Expanders - Apply equivalent subjects; Search modes – Proximity) (399)  3 SU aged OR SU (aged, 80 and over ) OR AB ( elderly OR senior OR seniors OR older adult OR older adults OR geriatric OR geriatrics OR old people OR older-age OR old age OR adult OR adults OR older people ) (Search Options: Limiters - Publication Date: 20230901- 20240931; Expanders - Apply equivalent subjects; Search modes – Proximity) (20448)  4 1 AND 2 AND 3 (29)  **5. CINAHL plus (via EBSCOhost) search**  Database: CINAHL plus with Full Text  1 SU perioperative period OR SU (perioperative care or perioperative nursing ) OR AB ( Pre-operati* OR Preoperati* OR Pre operati* OR Presurg* OR Presurg* OR Pre surg* OR prior to surgery OR before surgery OR Pre-operative Rehabilitat* OR Preoperative Rehabilitat* OR Preoperative Rehabilitat* OR Preoperative Conditioning* OR Preoperative Conditioning* OR Preoperative Conditioning* OR Preoperative training OR Preoperative training OR Pre operative training OR Pre-operative Exercise* OR Preoperative Exercise* OR Preoperative Exercise* OR Prehabilitat* OR Pre-habilitat* OR Pre habilitat* OR Prophylactic rehabilitat* OR Preventive rehabilitat* ) (Search Options: Limiters - Publication Date: 20230901- 20240931; Exclude MEDLINE records; Publication Type: Randomized Controlled Trials; Expanders - Apply equivalent subjects; Search modes – Proximity) (220)  2 SU postoperative cognitive complications OR SU ( delirium or acute confusion or confusion or disorientation ) OR AB ( postoperative cognitive complication* OR postoperative cognitive dysfunction* OR postoperative decline* OR postsurgical cognitive complication* OR post-surgical cognitive dysfunction* OR postoperative delirium OR postsurgical delirium OR acute confus* OR acute organic psychosyndrome* OR metabolic encephalopath* OR acute psycho-organic syndrome* OR clouded state* OR clouding of consciousness OR exogenous psycho* OR acute brain dysfunction OR toxic psycho* OR toxic confusion* OR toxic encephalopath* OR exogenous psycho* OR perioperative neurocognitive disorder* OR postoperative neurocognitive disorder* OR post-surgical neurocognitive disorder* OR PND OR POCD OR POD ) (Search Options: Limiters - Publication Date: 20230901- 20240931; Exclude MEDLINE records; Publication Type: Randomized Controlled Trials; Expanders - Apply equivalent subjects; Search modes – Proximity) (52)  3 SU aged OR SU (aged, 80 and over ) OR AB ( elderly OR senior OR seniors OR older adult OR older adults OR geriatric OR geriatrics OR old people OR older-age OR old age OR adult OR adults OR older people ) (Search Options: Limiters - Publication Date: 20230901- 20240931; Exclude MEDLINE records; Publication Type: Randomized Controlled Trials; Expanders - Apply equivalent subjects; Search modes – Proximity) (1969)  4 1 AND 2 AND 3 (7)  **6. PEDro database**  Abstract & Title (prehabilitation or preoperative) AND Subdiscipline (gerontology) AND Method (clinical trial) AND new records added since 06/09/2023 (0)  **7. CBM, CNKI, WANFANG database and VIP search**  1. Terms specific to prehabilitation  篇名,关键词,摘要(预处理) OR篇名,关键词,摘要(预康复) OR篇名,关键词,摘要(围术期康复)  2. Terms specific to surgery  篇名,关键词,摘要(手术)  3. Terms specific to aged  篇名,关键词,摘要(老年)  4. Terms specific to cognition  篇名,关键词,摘要(认知)  5. Published Year  时间范围(2023-至今)  6. 1 AND 2 AND 3 AND 4 AND 5 (CBM 80+CNKI 12+VIP 32+WANGFANG 29=153) |
| --- |

**Table 2.** Summary of findings.

| **Cognitive Prehabilitation for Older Adults Undergoing Elective Surgery** | | | |
| --- | --- | --- | --- |
| **Patient or population:** Older adults undergoing elective surgery **Settings:** hospital or home **Intervention:** cognitive prehabilitation **Comparison:** usual care | | | |
| **Outcomes** | **Effect** | **No of Participants (studies)** | **Quality of the evidence (GRADE)** |
| **POD incidence** | Vlisides and colleagues [44] did not find any significant difference between the experimental and control group (6/23 [26%] vs 5/29 [17%], P=0.507) (RR 1.51, 95% CI 0.53 to 4.34). Humeidan and colleagues [54] reported intervention may have little or no difference compared to control group (18/125 [14%] vs 29/126 [23%], P=0.08) (RR 0.63, 95% CI 0.37 to 1.07), and they found a reduction in POD incidence with their intervention on post hoc analysis (16/121 [13.2%] vs 29/126 [23%]; P=0.04) (RR 0.57, 95% CI 0.33 to 1). Greaves and colleagues [57] reported incident delirium outcomes that pre-operative CCT did not significantly associate with delirium following CABG surgery (OR=1.25, 95%CI=[0.30, 5.24], p=0.76). | 332 (3 studies) | ⊕⊝⊝⊝ **very low**^1,2,3^ |
| **dNCR incidence** | Saleh and colleagues [53] reported a significant difference in incidence of dNCR between the experimental group and control group (11/69 [16%] vs 26/72 [36%], P=0.007) (RR 0.44, 95% CI 0.24 to 0.82). Li and colleagues [55] reported similar results (6/36 [17%] vs 15/36 [42%], P<0.05) (RR 0.40, 95% CI 0.18 to 0.91). Wang and colleagues [56] found intervention resulted in a lower incidence of dNCR (6/50 [12%] vs 12/50 [24%]) (RR 0.50, 95% CI 0.20 to 1.23). | 313 (3 studies) | ⊕⊕⊝⊝ **low**^1,3^ |
| **Global cognition** | Li and colleagues [55] reported that experimental group was found to be associated with improved MoCA scores two weeks after intervention (27.28±1.69 vs 25.76±3.05, P<0.05) (MD 1.52, 95% CI 0.38 to 2.66). Wang and colleagues [56] reported that MMSE scores at postoperative day 7 between groups were significant (26.45±2.77 vs 23.05±1.56, P<0.05) (MD 3.40, 95% CI 2.52 to 4.28). Greaves and colleagues [57] found that there were no significant effects of computerized cognitive training on the change in ACE-III (t=0.25, DF=25.70, *p*=0.81, *d*=0.09) at discharge and in CANTAB at discharge: executive function (t=-0.08, DF=18.79, *p*=0.94, *d*=-0.03), psychomotor speed & attention (t=0.43, DF=22.95, *p*=0.67, *d*=0.16), spatial working memory (t=-1.76, DF=21.90, *p*=0.09, *d*=-0.66) | 201 (3 studies) | ⊕⊕⊝⊝ **low**^1,3,^ |
| GRADE Working Group grades of evidence **High quality:** Further research is very unlikely to change our confidence in the estimate of effect.  **Moderate quality:** Further research is likely to have an important impact on our confidence in the estimate of effect and may change the estimate. **Low quality:** Further research is very likely to have an important impact on our confidence in the estimate of effect and is likely to change the estimate. **Very low quality:** We are very uncertain about the estimate. | | | |
| ^1^ Downgraded due to imprecision caused by small sample size(the total number of patients included in all the trials was<400) ^2^ The direction of effect varied across the trials ^3^ Included studies were published in Chinese or English language | | | |

**Table 3.** Characteristics of excluded studies.

(Initial search)

| **No.** | **First Author** | **Year** | **Title** | **Main reason for exclusion** |
| --- | --- | --- | --- | --- |
| 1 | Olotu C | 2022 | The effect of delirium preventive measures on the occurrence of postoperative cognitive dysfunction in older adults undergoing cardiovascular surgery. The DelPOCD randomised controlled trial | Intervention: perioperative delirium prevention bundle |
| 2 | O'Gara BP | 2020 | Prevention of Early Postoperative Decline: A Randomized, Controlled Feasibility Trial of Perioperative Cognitive Training | Intervention: perioperative cognitive training |
| 3 | Zhang Lijuan | 2013 | Effects of early cognitive-behavioral therapy on postoperative cognitive dysfunction in older patients with hip fracture (in Chinese) | Intervention: multicomponent intervention |
| 4 | Lisann-Goldman LR | 2019 | Reducing Delirium and Improving Patient Satisfaction with a Perioperative Mindfulness Intervention: A Mixed-Methods Pilot Study | Intervention: perioperative mindfulness intervention |
| 5 | Deeken F | 2022 | Outcomes of a Delirium Prevention Program in Older Persons After Elective Surgery: A Stepped-Wedge Cluster Randomized Clinical Trial | Intervention: multifaceted delirium prevention intervention |
| 6 | Xia Yun | 2015 | The effect of transcutaneous electrical acupoint stimulation combined with cognitive training on cognitive function in patients after laparoscopic operation (in Chinese) | Intervention: multicomponent intervention |
| 7 | Zhu Yuqing | 2020 | Effects of predictive psychological nursing in operating room on negative emotions, cognitive function and health status of older patients after operation under general anesthesia (in Chinese) | Intervention: perioperative multicomponent intervention |
| 8 | Chen CC | 2017 | Effect of a Modified Hospital Elder Life Program on Delirium and Length of Hospital Stay in Patients Undergoing Abdominal Surgery: A Cluster Randomized Clinical Trial | Intervention: postoperative multicomponent care |
| 9 | Atkins KJ | 2021 | Preventing Delirium and Promoting Long-Term Brain Health: A Clinical Trial Design for the Perioperative Cognitive Enhancement (PROTECT) Trial | Intervention: perioperative multicomponent intervention |
| 10 | Wang Shuangyan | 2021 | To investigate the effect of prescient psychological nursing in the operating room on postoperative rehabilitation and cognitive function of elderly patients under general anesthesia (in Chinese) | Intervention: perioperative multicomponent intervention |
| 11 | Guo Min | 2021 | Effects of predictive psychological nursing in operating room on postoperative rehabilitation and cognitive function in older patients with general anesthesia (in Chinese) | Intervention: perioperative multicomponent intervention |
| 12 | Watne LO | 2014 | The effect of a pre- and postoperative orthogeriatric service on cognitive function in patients with hip fracture: randomized controlled trial (Oslo Orthogeriatric Trial) | Intervention: perioperative multicomponent care |
| 13 | Unal N | 2022 | Evaluation of the effectiveness of delirium prevention care protocol for the patients with hip fracture: A randomised controlled study | Intervention: perioperative delirium prevention care bundle |
| 14 | Marcantonio ER | 2001 | Reducing delirium after hip fracture: a randomized trial | Intervention: perioperative geriatrics consultation |
| 15 | Hempenius L | 2016 | Long Term Outcomes of a Geriatric Liaison Intervention in Frail Elderly Cancer Patients | Intervention: geriatric liaison intervention (perioperative multicomponent care) |
| 16 | Hempenius L | 2013 | Outcomes of a Geriatric Liaison Intervention to Prevent the Development of Postoperative Delirium in Frail Elderly Cancer Patients: Report on a Multicentre, Randomized, Controlled Trial | Intervention: geriatric liaison intervention (perioperative multicomponent care) |
| 17 | Guo Y | 2016 | Impact of multicomponent, nonpharmacologic interventions on perioperative cortisol and melatonin levels and postoperative delirium in elderly oral cancer patients | Intervention: perioperative multicomponent care |
| 18 | Pan Hongli | 2021 | Effect of predictive psychological nursing in operating room on elderly patients undergoing general anesthesia (in Chinese) | Intervention: perioperative multicomponent intervention |
| 19 | Jiang Huili | 2014 | Effects of electroacupuncture preconditioning on postoperative cognitive dysfunction in elderly patients undergoing joint replacement (in Chinese) | Intervention: electroacupuncture preconditioning (not cognition-oriented treatments) |
| 20 | Fu Tingyou | 2019 | Effects of hyperbaric oxygen preconditioning on the cognitive function of elderly patients with lower extremity fracture after surgery (in Chinese) | Intervention: hyperbaric oxygen preconditioning (not cognition-oriented treatments) |
| 21 | Mouchoux C | 2011 | Impact of a multifaceted program to prevent postoperative delirium in the elderly: the CONFUCIUS stepped wedge protocol | Intervention: perioperative multifaceted delirium prevention care |
| 22 | Zhao Feiyi | 2018 | The effect of electroacupuncture preconditioning on cognitive impairments following knee replacement among elderly: A randomized controlled trial (in Chinese) | Intervention: electroacupuncture preconditioning (not cognition-oriented treatments) |
| 23 | Liu Peirong | 2017 | Effect of electroacupuncture at “four close’’ points on cognitive function in elderly patients with hip replacement (in Chinese) | Intervention: perioperative electroacupuncture therapy (not cognition-oriented treatments) |
| 24 | Liu Peirong | 2018 | Effects of Electroacupuncture on Immune Function, Adrenal Stress and Cognitive Function in Elder1y Patients Undergoing Hip Replacement (in Chinese) | Intervention: perioperative electroacupuncture therapy (not cognition-oriented treatments) |
| 25 | Kim S | 2021 | A pilot study of aquatic prehabilitation in adults with knee osteoarthritis undergoing total knee arthroplasty - short term outcome | Intervention: aquatic prehabilitation (not cognition-oriented treatments) |
| 26 | Xi Lijuan | 2021 | Effect of Perioperative Transcutaneous Electrical Acupoint Stimulation on the Early Postoperative Rehabilitation for Elderly Patients with Gastrointestinal Tumor (in Chinese) | Intervention: perioperative transcutaneous electrical acupoint stimulation (not cognition-oriented treatments) |
| 27 | Li Jia | 2021 | To explore the effect of predictive psychological nursing in operating room on postoperative rehabilitation and cognitive function in older patients with general anesthesia (in Chinese) | Intervention: perioperative psychological nursing |
| 28 | Xi L | 2021 | Transcutaneous electrical acupoint stimulation for postoperative cognitive dysfunction in geriatric patients with gastrointestinal tumor: a randomized controlled trial | Intervention: perioperative transcutaneous electrical acupoint stimulation (not cognition-oriented treatments) |
| 29 | Trubnikova OA | 2021 | Beneficial Effects of a Short Course of Physical Prehabilitation on Neurophysiological Functioning and Neurovascular Biomarkers in Patients Undergoing Coronary Artery Bypass Grafting | Intervention: physical prehabilitation for patients aged 45-70 |
| 30 | Edwards C | 2020 | A pilot study to evaluate the effects of acupuncture on post-operative cognitive dysfunction (POCD) in older adults: a randomized, controlled clinical trial | Conference abstracts |
| 31 | Zhu Yulin | 2012 | Effects of hyperbaric oxygen preconditioning on postoperative cognitive function in older patients undergoing hip replacement surgery (in Chinese) | Intervention: hyperbaric oxygen preconditioning (not cognition-oriented treatments) |
| 32 | Zhang Huiling | 2021 | Effects of predictive psychological nursing in operating room on negative emotions, cognitive function and health status of older patients after operation under general anesthesia (in Chinese) | Intervention: perioperative psychological nursing |
| 33 | Stenvall M | 2007 | A multidisciplinary, multifactorial intervention program reduces postoperative falls and injuries after femoral neck fracture | Intervention: postoperative multifactorial intervention program |
| 34 | Zhang Q | 2017 | Effects of preconditioning of electro-acupuncture on postoperative cognitive dysfunction in elderly: A prospective, randomized, controlled trial | Intervention: one-session electroacupuncture preconditioning (not cognition-oriented treatments) |
| 35 | Duan Chongzhen | 2021 | Effects of percutaneous acupoint electrical stimulation pretreatment on postoperative cognitive function and inflammatory factors in older patients (in Chinese) | Intervention: one-session electroacupuncture preconditioning (not cognition-oriented treatments) |
| 36 | Meybohm P | 2013 | Postoperative neurocognitive dysfunction in patients undergoing cardiac surgery after remote ischemic preconditioning: a double-blind randomized controlled pilot study | Intervention: one-session remote ischemic preconditioning (not cognition-oriented treatments) |
| 37 | Hudetz JA | 2015 | Remote ischemic preconditioning prevents deterioration of short-term postoperative cognitive function after cardiac surgery using cardiopulmonary bypass: results of a pilot investigation | Intervention: one-session remote ischemic preconditioning (not cognition-oriented treatments) |
| 38 | Wei H | 2022 | Transcutaneous Electrical Acupoint Stimulation Improves Postoperative Cognitive Function in Senior Patients Undergoing Video-Assisted Thoracoscopic Surgery: A Randomized Controlled Trial | Intervention: one-session electroacupuncture preconditioning (not cognition-oriented treatments) |
| 39 | Liu T | 2021 | Effects of Transcutaneous Electrical Acupoint Stimulation on Postoperative Cognitive Decline in Elderly Patients: A Pilot Study | Intervention: one-session electroacupuncture preconditioning (not cognition-oriented treatments) |
| 40 | Lin SY | 2013 | Impacts of the different frequencies of electroacupunctrue on cognitive function in patients after abdominal operation under compound anesthesia of acupuncture and drugs | Intervention: one-session electroacupuncture preconditioning (not cognition-oriented treatments) |
| 41 | Li XZ | 2016 | Effects of Scalp Acupuncture on Serum NSE and S-100β Concentrations and Postoperative Cognitive Function of Elderly Patients Undergoing Hip Replacement | Intervention: one-session scalp acupuncture (not cognition-oriented treatments) during the operation |
| 42 | Gao XQ | 2012 | Effects of electroacupuncture assistant general anesthesia on postoperative cognitive dysfunction of aged patients | Intervention: one-session electroacupuncture preconditioning (not cognition-oriented treatments) |
| 43 | Gao F | 2018 | Transcutaneous electrical acupoint stimulation for prevention of postoperative delirium in geriatric patients with silent lacunar infarction: a preliminary study | Intervention: one-session transcutaneous electrical acupoint stimulation (not cognition-oriented treatments) |
| 44 | Bosco G | 2014 | Preconditioning with hyperbaric oxygen in pancreaticoduodenectomy: a randomized double-blind pilot study | Intervention: one-session hyperbaric oxygen preconditioning (not cognition-oriented treatments) |
| 45 | Zhou Xiang | 2014 | Effect of lower extremity ischemic preconditioning on postoperative cognitive function in elderly patients undergoing radical resection of rectal carcinoma (in Chinese) | Intervention: one-session lower extremity ischemic preconditioning (not cognition-oriented treatments) |
| 46 | Zhou Biao | 2018 | Effect of remote ischemic preconditioning on postoperative cognition in elderly patients undergoing liver cancer surgery (in Chinese) | Intervention: one-session remote ischemic preconditioning (not cognition-oriented treatments) |
| 47 | Yang Tao | 2021 | The effect of transcutaneous acupoint electrical stimulation combined with dexmedetomidine on the cognitive function of elderly patients after hip surgery (in Chinese) | Intervention: one-session transcutaneous acupoint electrical stimulation (not cognition-oriented treatments) |
| 48 | Wang Haili | 2021 | Effect of multi-mode pretreatment on postoperative cognitive dysfunction in elderly patients with lumbar internal fixation (in Chinese) | Intervention: Peri-anesthesia period multimodal treatment |
| 49 | Wang Dongdong | 2016 | Clinical observation of transcutaneous electrical acupoint stimulation in the treatment of cognitive dysfunction after abdominal surgery under general anesthesia in older patients (in Chinese) | Intervention: one-session transcutaneous electrical acupoint stimulation (not cognition-oriented treatments) |
| 50 | Guo Peng | 2017 | Effects of distal limb ischemic preconditioning on postoperative cognitive dysfunction in older orthopedic patients (in Chinese) | Intervention: one-session remote ischemic preconditioning (not cognition-oriented treatments) |
| 51 | Janssen TL | 2020 | Long-term outcomes of major abdominal surgery and postoperative delirium after multimodal prehabilitation of older patients | Study design: an uncontrolled before-and-after study |
| 52 | Janssen TL | 2019 | Multimodal prehabilitation to reduce the incidence of delirium and other adverse events in elderly patients undergoing elective major abdominal surgery: An uncontrolled before-and-after study | Study design: an uncontrolled before-and-after study |
| 53 | Pernik MN | 2021 | Perioperative Optimization of Senior Health in Spine Surgery: Impact on Postoperative Delirium. | Study design: retrospectively study compared to a matched historical control cohort |
| 54 | Lee J | 2013 | Perioperative psycho-educational intervention can reduce postoperative delirium in patients after cardiac surgery: a pilot study | Study design: a comparative retrospective study |
| 55 | Kratz T | 2015 | Preventing postoperative delirium: A prospective intervention with psychogeriatric liaison on surgical wards in a general hospital | Study design: an open non-randomised clinical study |
| 56 | Wang Li | 2017 | Pain cognitive intervention and adverse reactions in perioperative elderly patients undergoing total knee replacement (in Chinese) | Objective: to investigate effect of cognition intervention on pain (mainly involving pain propaganda and education) in perioperative elderly patients undergoing total knee replacement |
| 57 | Siggeirsdottir K | 2005 | Short hospital stay augmented with education and home-based rehabilitation improves function and quality of life after hip replacement: randomized study of 50 patients with 6 months of follow-up | Objective: to investigate effect of perioperative multifaceted intervention on function and quality of life after hip replacement |
| 58 | Pour AE | 2007 | Minimally invasive hip arthroplasty: what role does patient preconditioning play? | Objective: investigate the role of perioperative multifaceted intervention on the outcome of total hip arthroplasty performed through a small incision |
| 59 | Karlsson E | 2021 | Well begun is half done: Preoperative physical performance & home-based exercise in older adults undergoing abdominal cancer surgery | Dissertation abstracts |
| 60 | Chikamori F | 2004 | Perioperative music therapy with a key-lighting keyboard system in elderly patients undergoing digestive tract surgery. | Objective: to investigate effect of perioperative music therapy with a key-lighting keyboard system on postoperative hemodynamics and perioperative mental functioning in elderly patients underwent digestive tract surgery |
| 61 | Lisann L | 2016 | Remind: Reducing delirium and improving patient satisfaction with a perioperative mindfulness intervention | Conference abstracts |
| 62 | Mouchoux C | 2010 | Methodology for assessing the impact of a multidisciplinary prevention program to prevent postoperative delirium in the elderly | Conference abstracts |
| 63 | Rengel KF | 2021 | A randomised pilot trial of combined cognitive and physical exercise prehabilitation to improve outcomes in surgical patients | Population: included participants aged 18 years and older |
| 64 | Humeidan ML | 2015 | Perioperative Cognitive Protection-Cognitive Exercise and Cognitive Reserve (The Neurobics Trial): A Single-blind Randomized Trial | Commentary without data |

(updated search at September 6, 2023)

| **No.** | **First Author** | **Year** | **Title** | **Main reason for exclusion** |
| --- | --- | --- | --- | --- |
| 1 | Alvarez EA | 2023 | Non-pharmacological prevention of postoperative delirium by occupational therapy teams: A randomized clinical trial | Intervention: postoperative occupational therapy |
| 2 | Xue Ling | 2023 | Cognitive behavioral therapy combined with escitalopram to alleviate perioperative anxiety in elderly patients with colorectal cancer and its effect on postoperative delirium (in Chinese) | Intervention: multicomponent intervention |
| 3 | Cai Lingling | 2022 | Effect of predictive psychological nursing in operating room on senile patients under general anesthesia after operation (in Chinese) | Intervention: perioperative multicomponent intervention |
| 4 | Chen Qiong | 2023 | Application of different cognitive function training methods in postoperative nursing of elderly patients with hip replacement (in Chinese) | Intervention: perioperative cognitive function training |

(updated search at September 5, 2024).

| **No.** | **First Author** | **Year** | **Title** | **Main reason for exclusion** |
| --- | --- | --- | --- | --- |
| 1 | Jiang Y | 2024 | Cognitive Training for Reduction of Delirium in Patients Undergoing Cardiac Surgery: A Randomized Clinical Trial | Population: included participants aged 18 years and older |
| 2 | Chen CY | 2024 | Effectiveness of Roy Adaptation Model-Based Cognitive Stimulation Therapy in Elderly Patients with Non-Small Cell Lung Cancer Undergoing Curative Resection | Intervention: perioperative cognitive stimulation |
| 3 | Ros-Nebot B | 2024 | Cognitive Training to Reduce Memory Disturbance Associated With Postoperative Cognitive Impairment After Elective Noncardiac Surgery: An Experimental Study | Population: included participants under the age of 60 |

**Figure 1.** Cognitive prehabilitation effects on the incidence of POD.


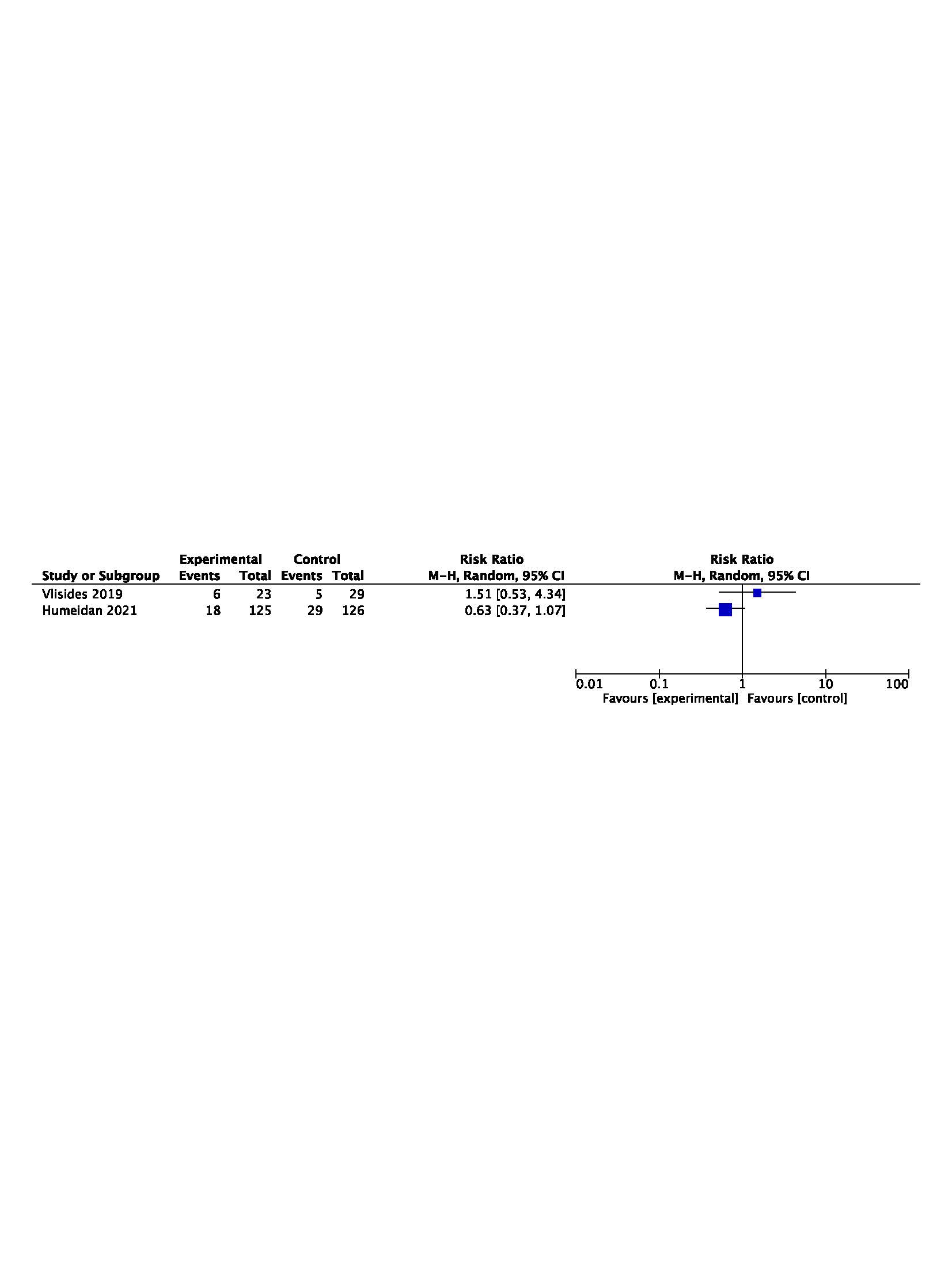


**Figure 2.** Cognitive prehabilitation effects on the incidence of dNCR.


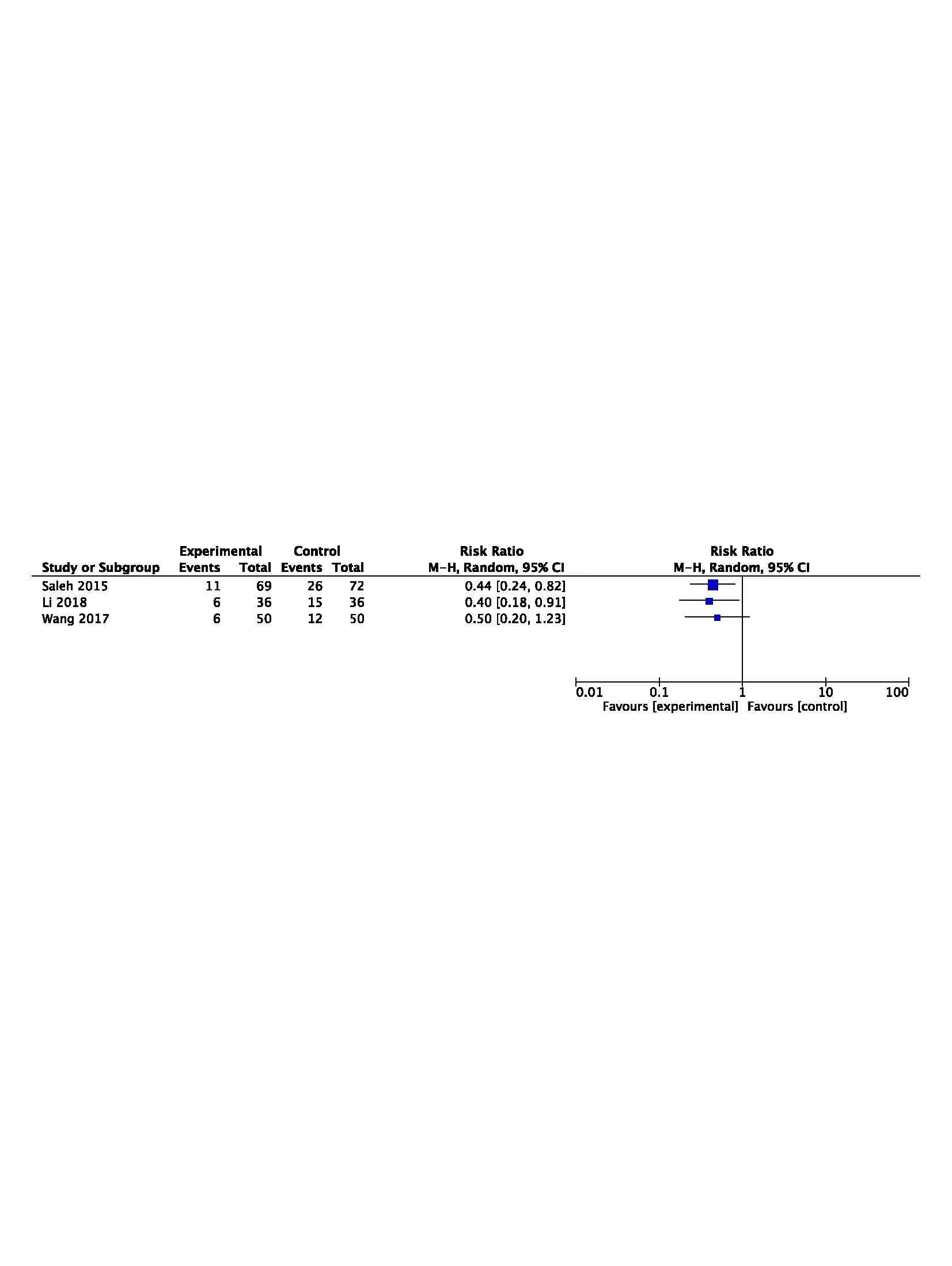


**Figure 3.** Global cognitive function: MoCA.


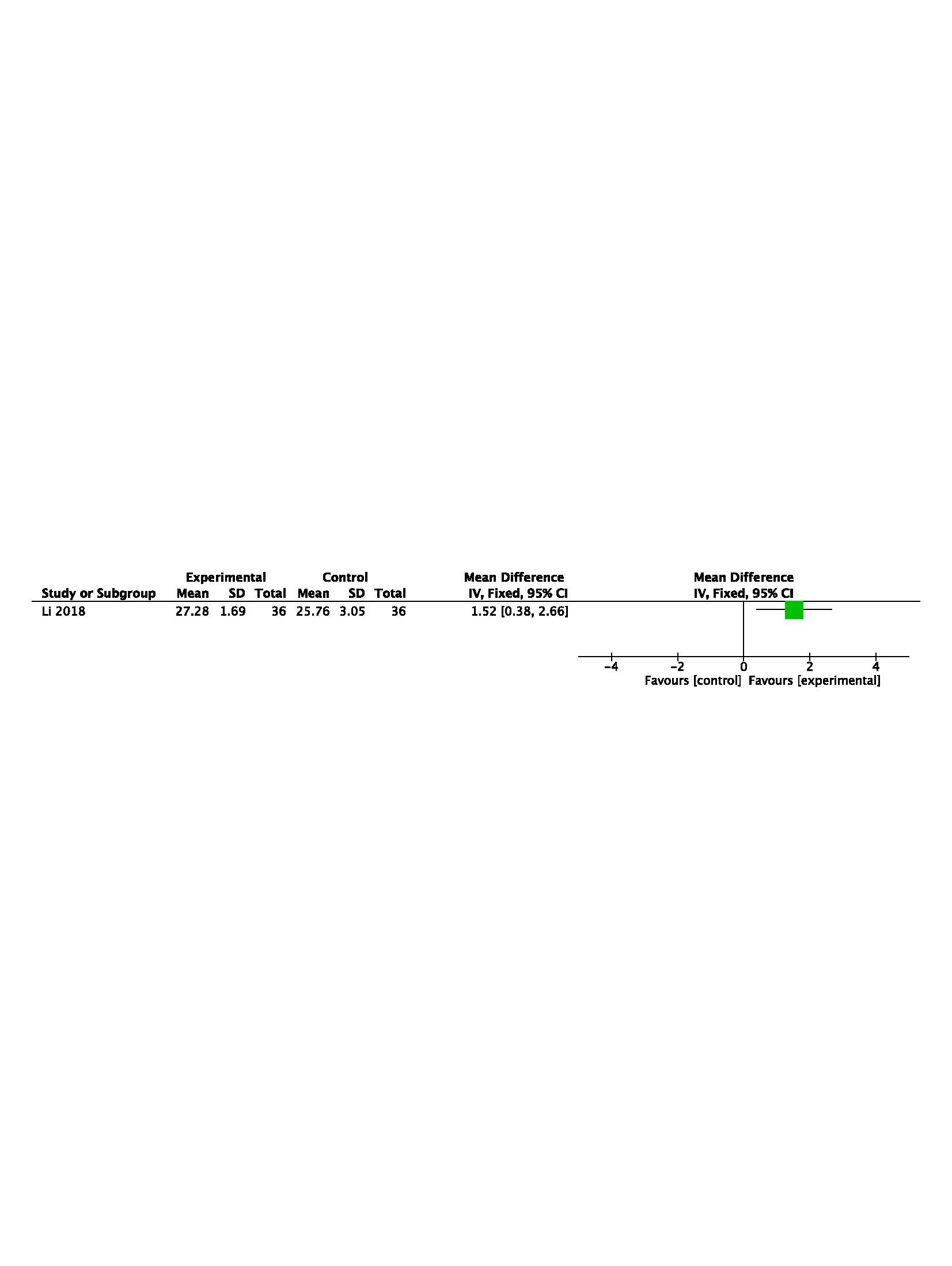


**Figure 4.** Global cognitive function: MMSE.


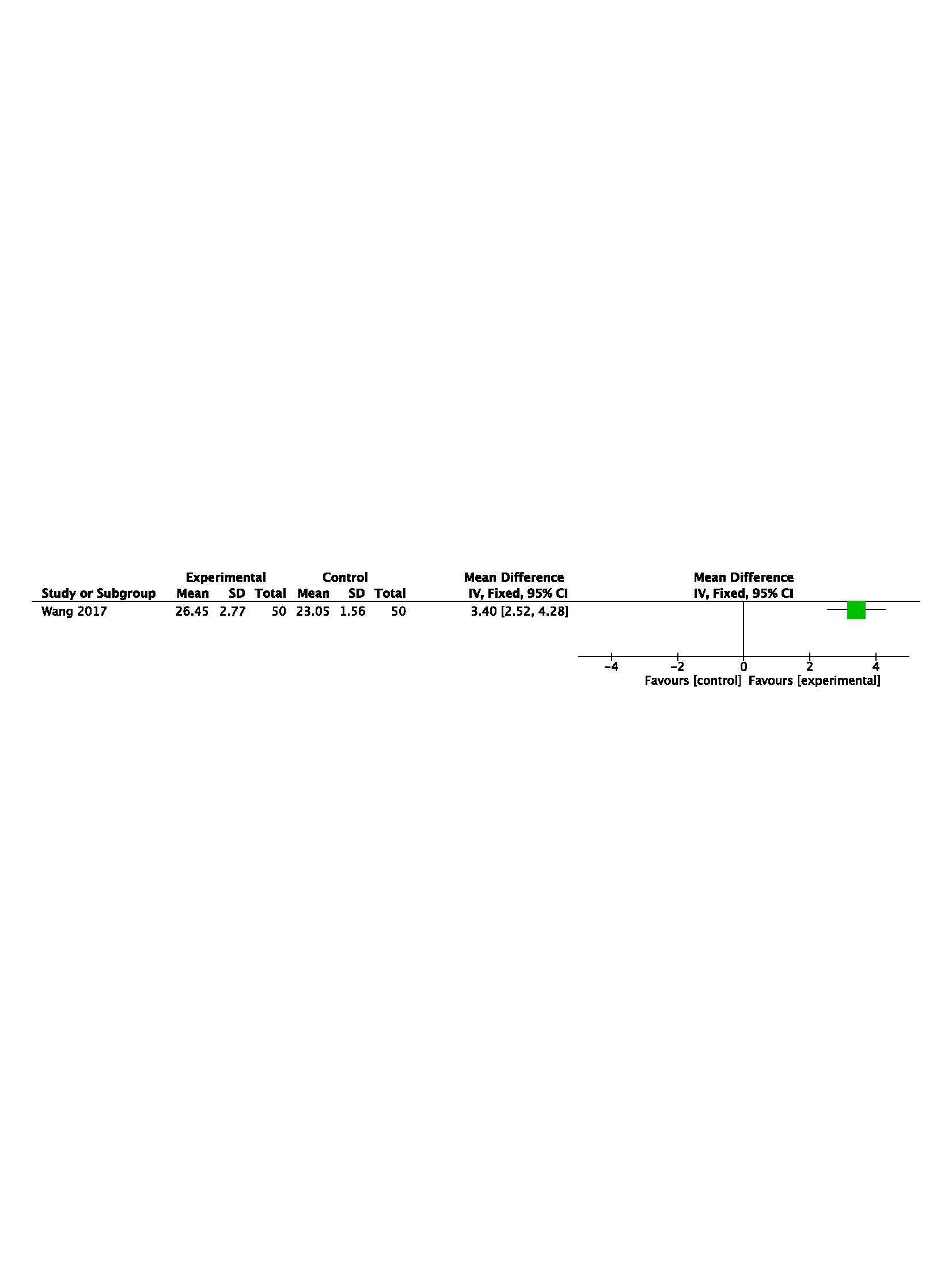


**Table 4.** Effects of cognitive prehabilitation: other outcomes

| Length of hospital stay: Three studies reported hospital LOS [44,53,54]. Vlisides and colleagues [44] reported a hospital LOS of 6.4 days for participants in the control group and 6.8 days for those in the cognitive prehabilitation group (P = 0.696). However, they did not report the standard deviation for each study arm. Saleh and colleagues [53] reported that cognitive prehabilitation may reduce the LOS by approximately 1 day compared to that in the usual care control group (MD -1.05, 95% CI -1.58 to -0.52; 141 participants; Supplementary Figure 5). Humeidan and colleagues [54] reported the duration of hospital stay as the median number of days within the interquartile range (IQR). For the cognitive prehabilitation group, the LOS was 4 days (IQR 3–6), compared to 4 days (IQR 3–6) for the normal daily activity control group (P = 0.55).  Postoperative complications: One study reported data presented as the number (%) of postoperative complications [53]. Differences in the incidence of neurological complications (RR 0.52, 95% CI 0.05 to 5.62; 141 participants; Supplementary Figure 6), respiratory complications (RR 1.57, 95% CI 0.27 to 9.08; 141 participants; Supplementary Figure 7), cardiovascular complications (RR 2.09, 95% CI 0.19 to 22.50; 141 participants; Supplementary Figure 8), infection (RR 0.78, 95% CI 0.29 to 2.14; 141 participants; Supplementary Figure 9), and intensive care unit stay for >24 h (RR 0.70, 95% CI 0.12 to 4.04; 141 participants; Supplementary Figure 10) were not statistically significant between the two groups.  ADLs: Only one study reported on ADLs [56]. It was unclear as to whether cognitive prehabilitation improved scores on the Barthel Index (MD -0.46, 95% CI -1.98 to 1.06; 100 participants; Supplementary Figure 11) immediately before surgery, although it may affect the capacity for ADLs 7 days postoperatively compared to usual care (MD 11.13, 95% CI 8.70 to 13.56; 100 participants; Supplementary Figure 12).  Patient acceptability and compliance: Acceptability and compliance with cognitive training before surgery were mentioned in two studies that provided computer-based training programs in a home setting [44,54]. Vlisides and colleagues [44] reported that during the course of the patient recruitment process, the most common nonmedical reasons for declining participation were lack of computer access (n = 19), time commitment (n = 9), and feeling overwhelmed preoperatively (n = 9). Additionally, the proportion of participants who withdrew from the trial was significantly greater in the prehabilitation group (5/30, 16.7%) than in the control group (0/31, 0%, P = 0.024), with reasons such as anxiety (n = 3), technical issues (n = 1), and not finding the training enjoyable (n = 1). Regarding intervention compliance, 48% of the patients in the intervention group opted out of training once at home, with the most common reasons being feeling overwhelmed (n = 4) and technical computer difficulties (n = 3). Only 17% of patients were able to complete the prescribed 7 days of training. For those who engaged in the training exercises, the median number of days of training was 6 (IQR 5–7), and the length of daily training sessions was 20 min (IQR 11–23). Humeidan and colleagues [54] defined minimum compliance with an intervention as participation in certain brain exercises. A total of 121 of 125 patients (96.8%) in the intervention group met the definition of minimum compliance. However, the duration of preoperative cognitive exercise varied widely (ranging from 0 to 32.5 h), with only 11 of 125 patients (8.8%) completing the goal of a 10-h training per study protocol. The median duration of preoperative cognitive exercise was 4.6 h (IQR 1.3–7.4).  Adverse events: No serious adverse events or unintended effects were reported during the trial by Humeidan and colleagues [54]. The other four studies did not mention adverse events. |
| --- |

**Figure 5.** Length of hospital stay.


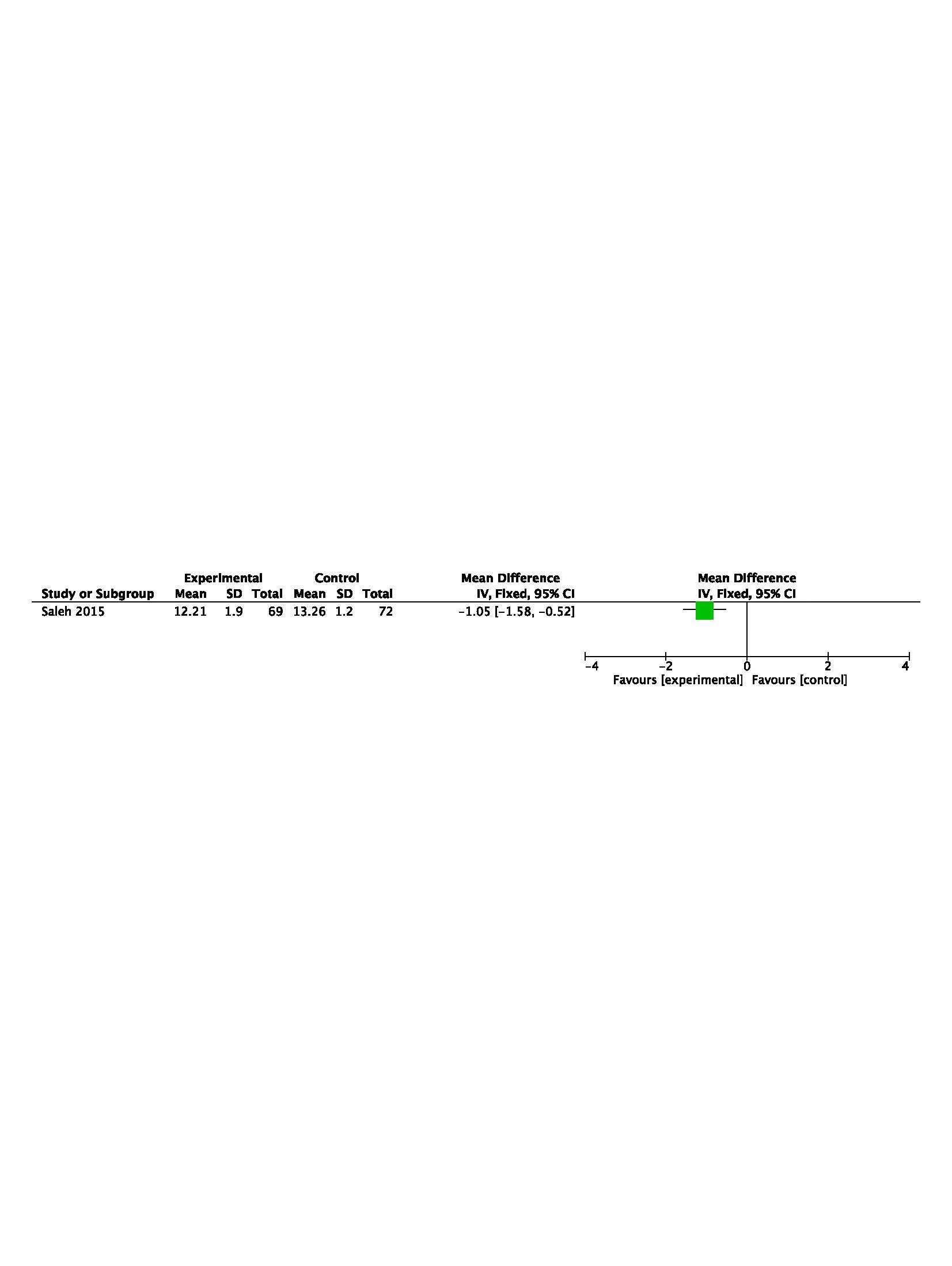


**Figure 6.** Incidence of neurological complications.


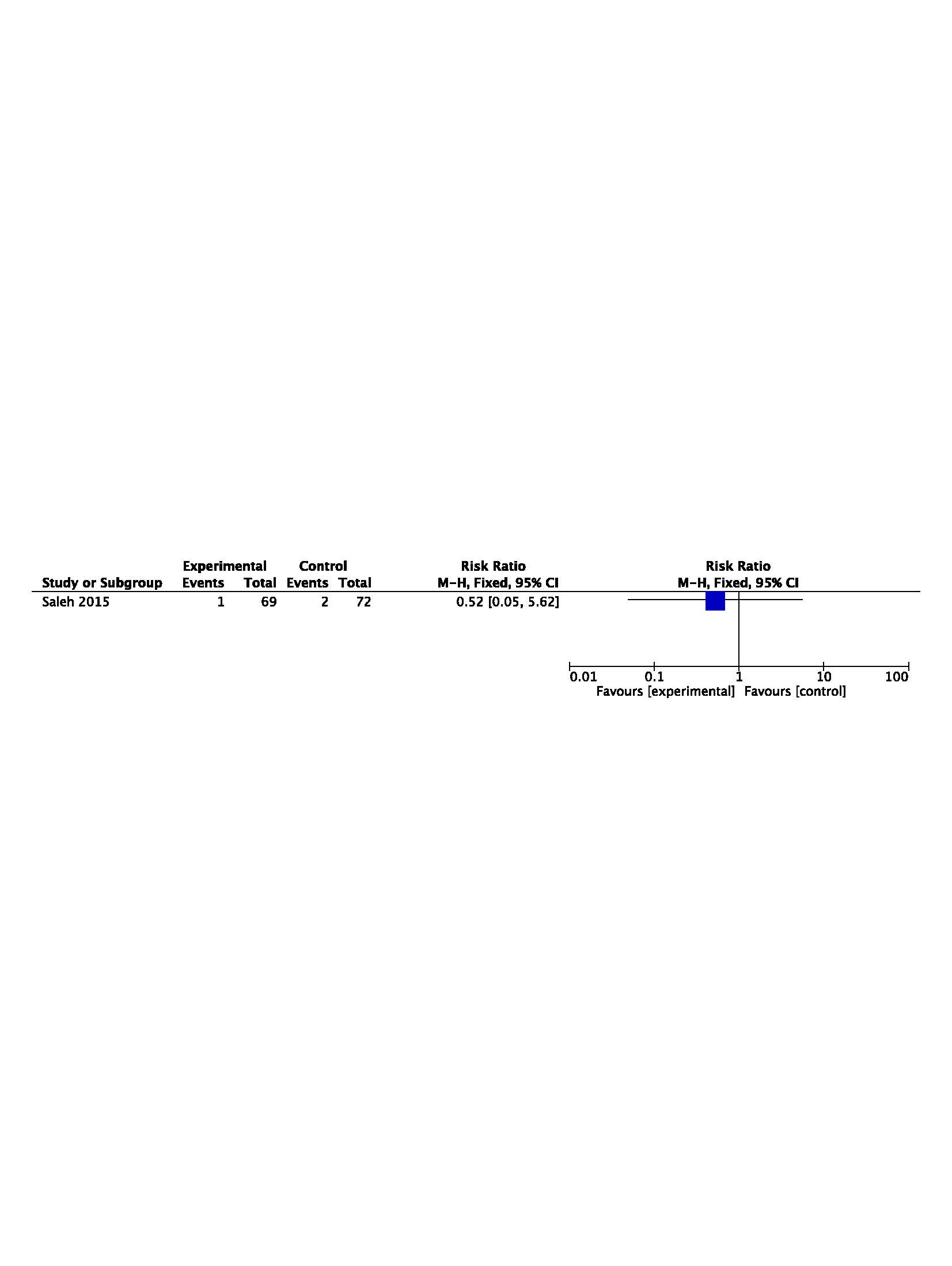


**Figure 7.** Incidence of respiratory complications.


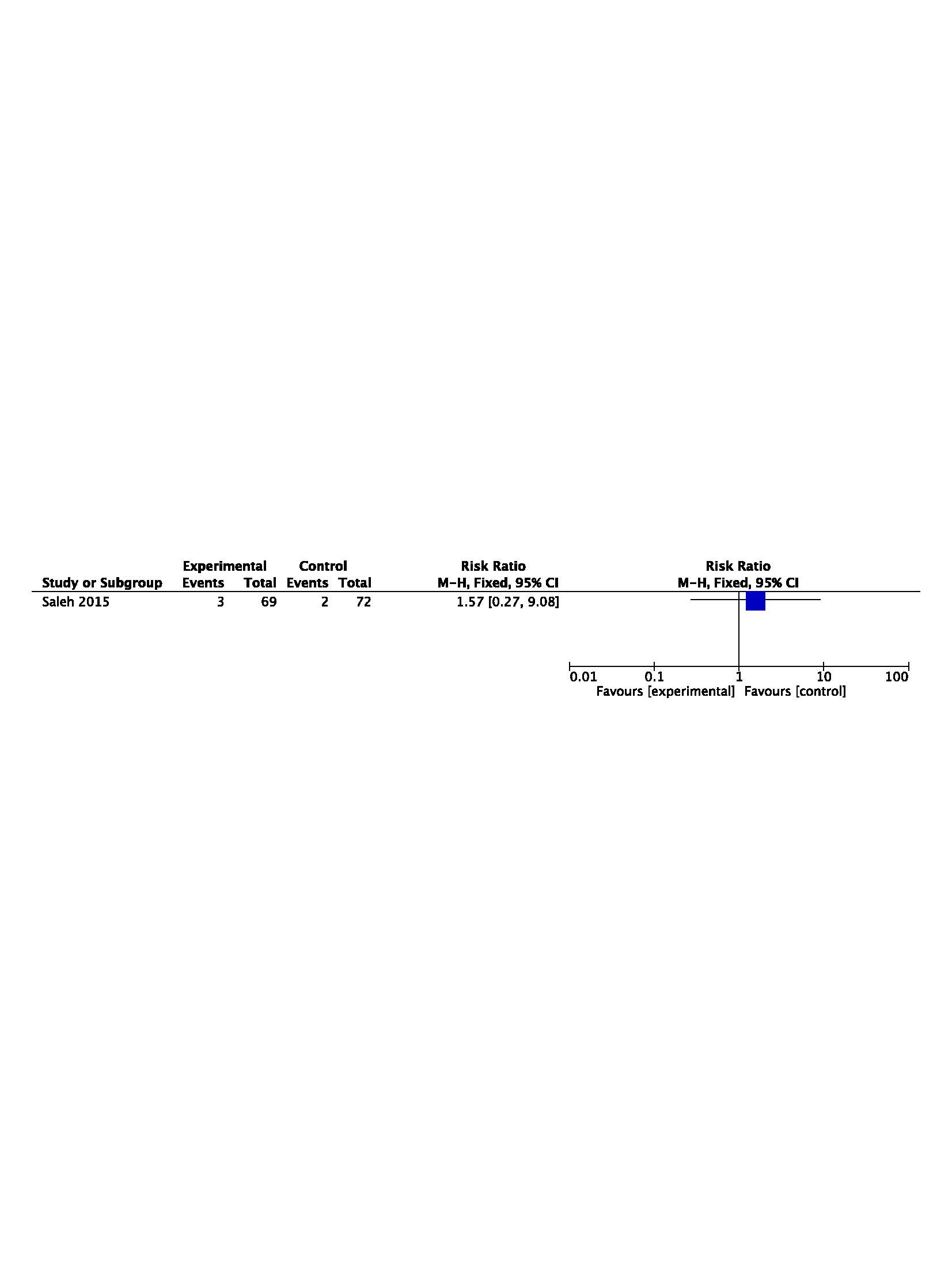


**Figure 8.** Incidence of cardiovascular complications.


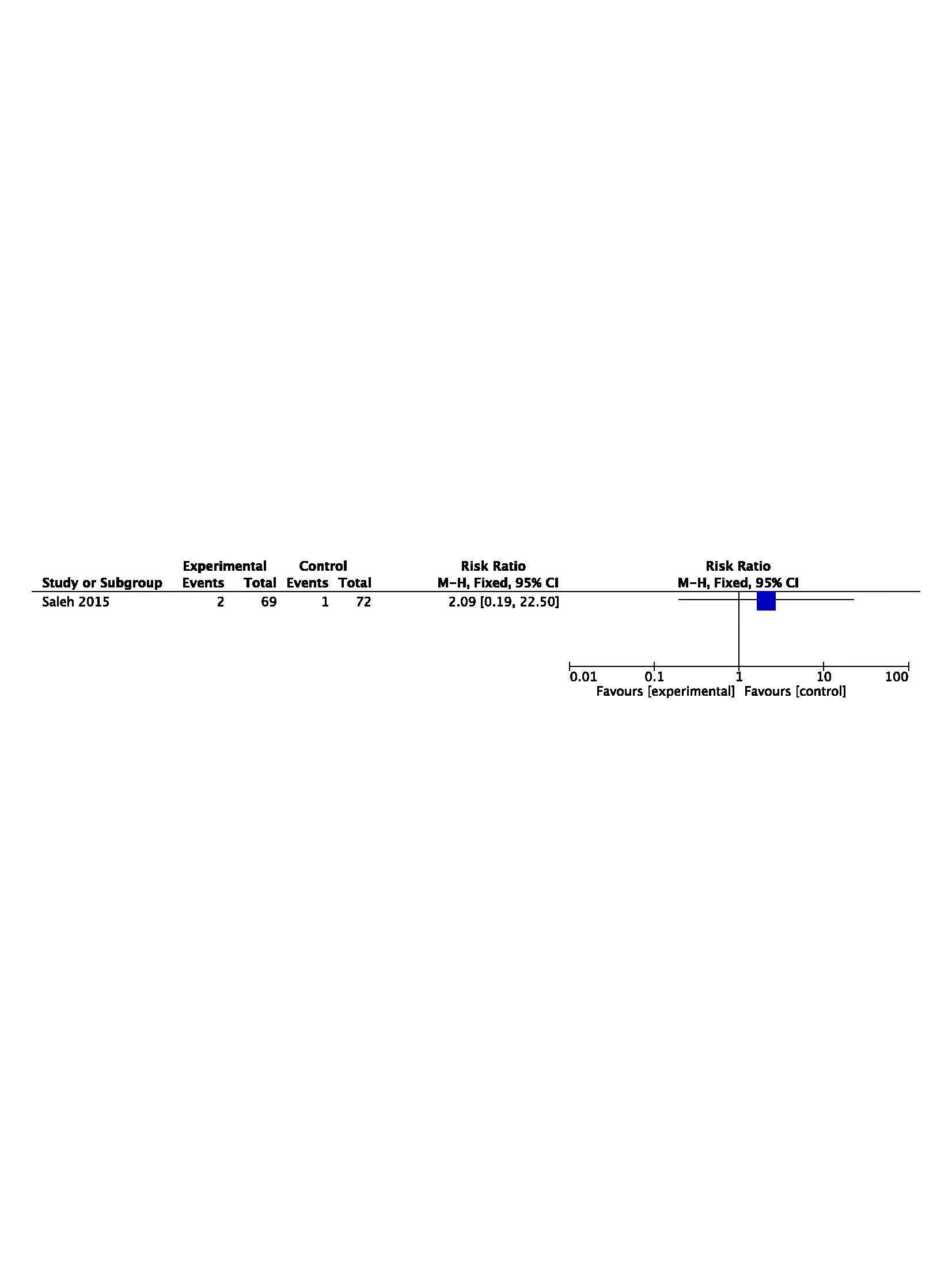


**Figure 9** Incidence of infection.


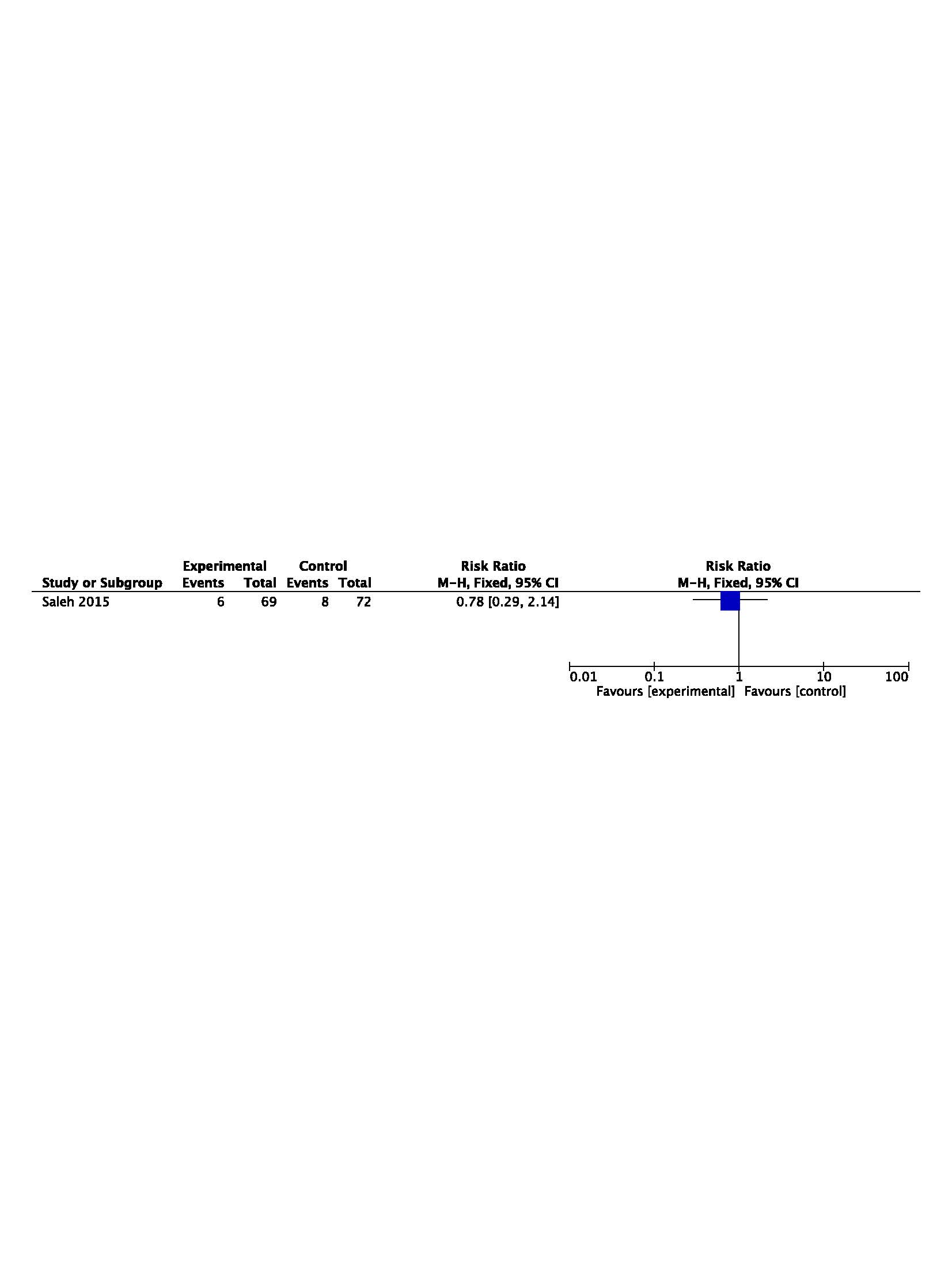


**Figure 10.** Incidence of intensive care unit stay for >24 h.


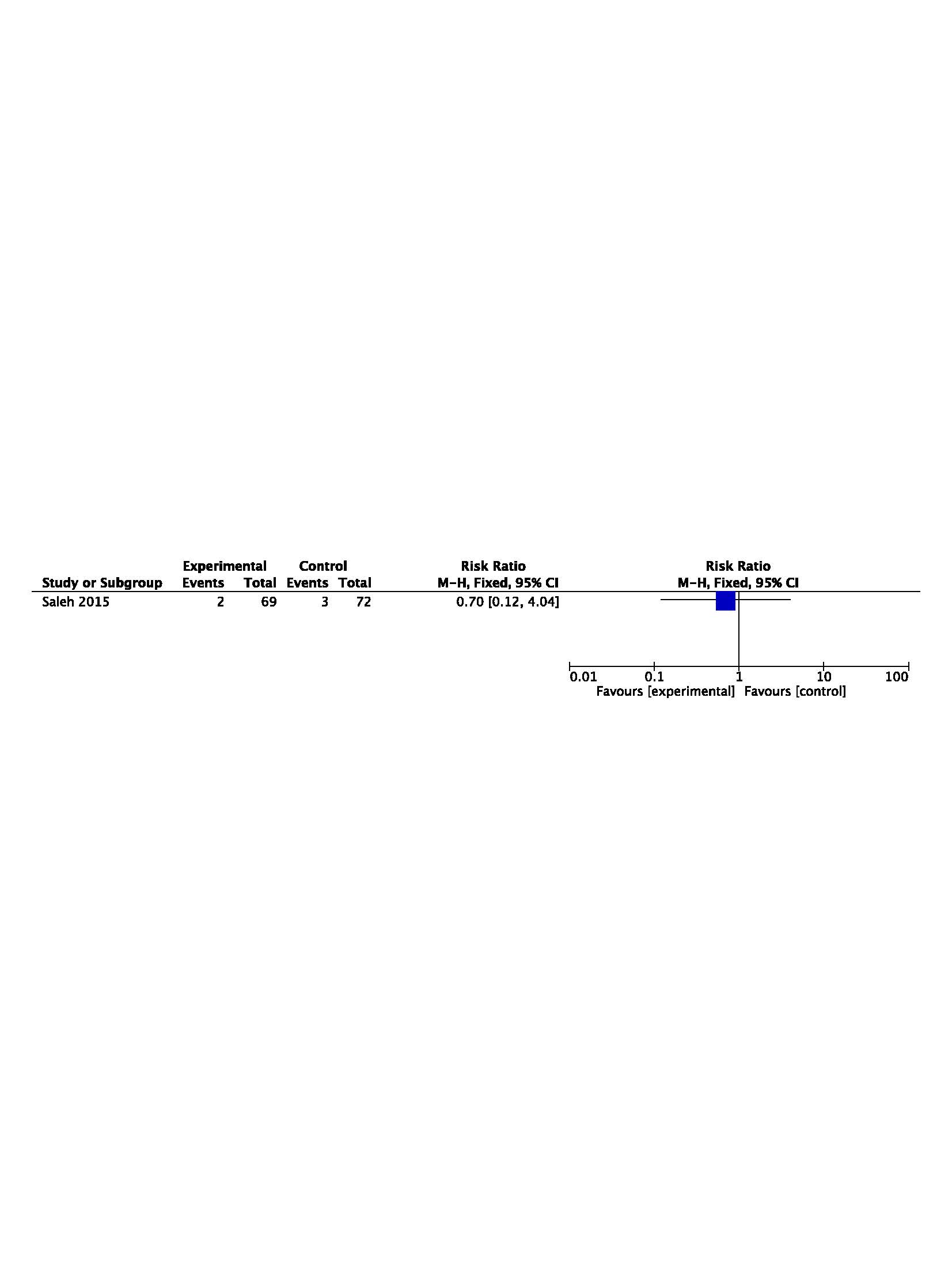


**Figure 11.** ADL immediately before surgery.


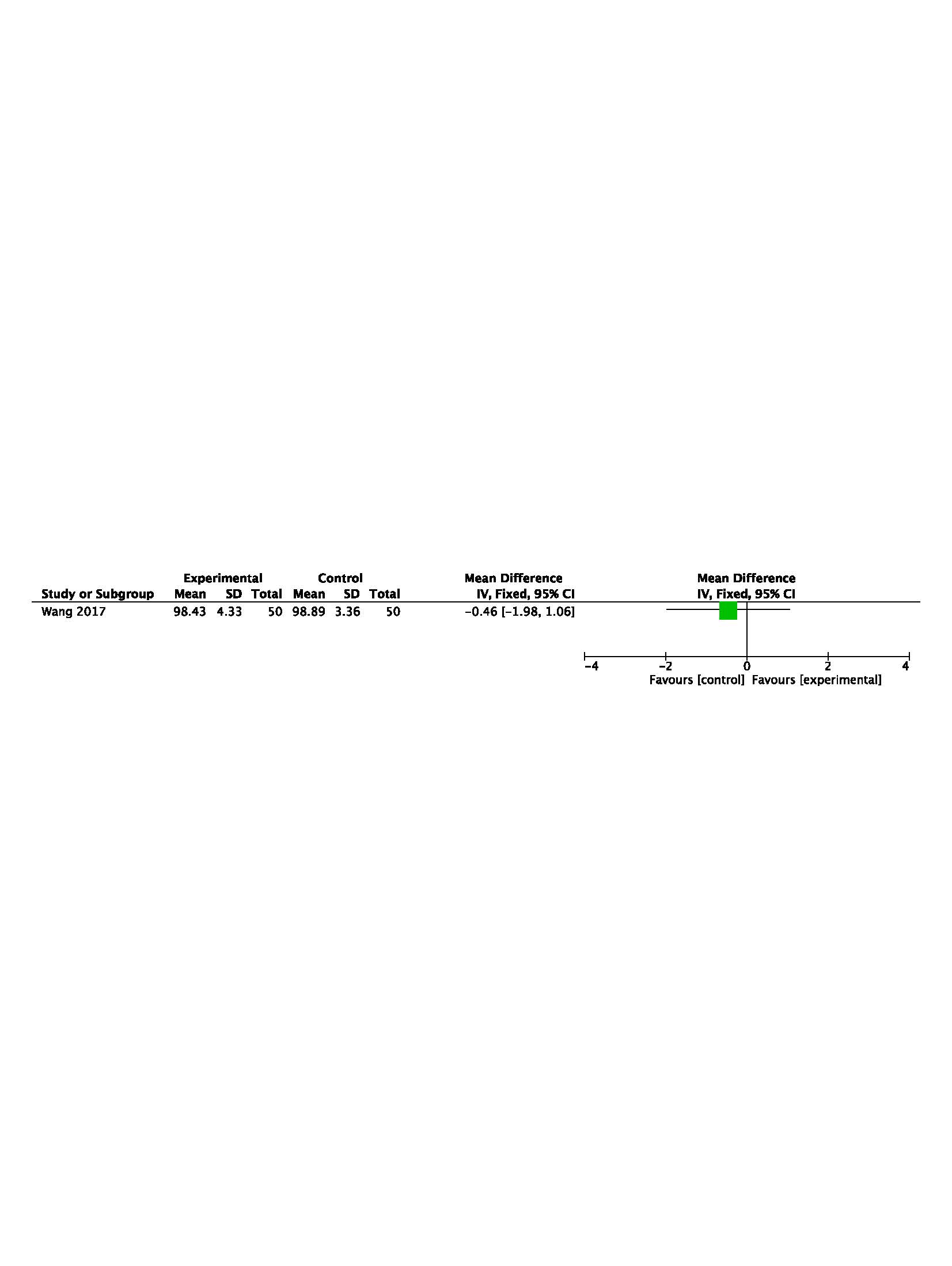


**Figure 12.** ADL at 7 days after surgery.


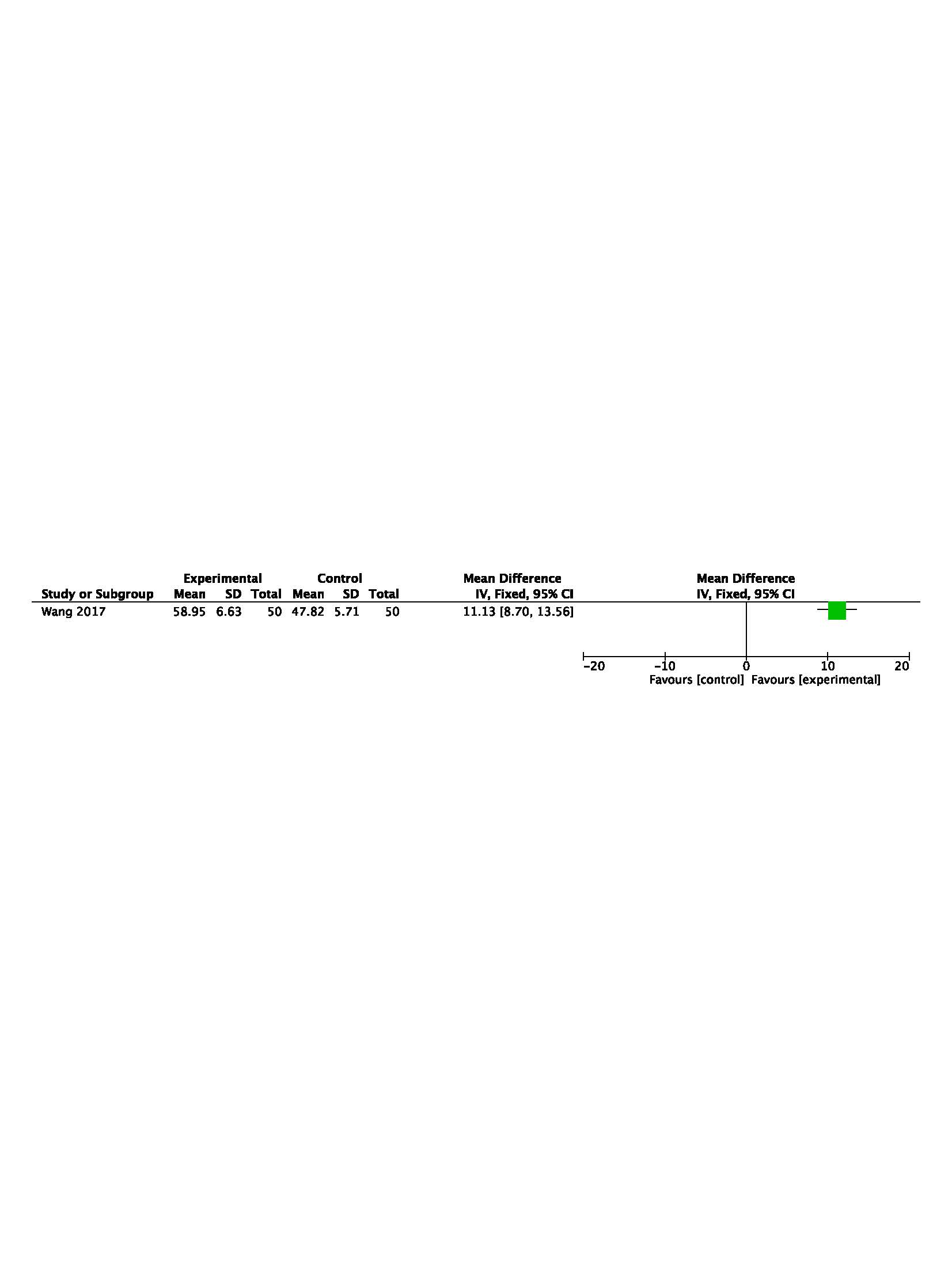


**Table 5.** Differences between protocol and review.

| Search methods:  The patient-intervention-comparison-outcome-study design (PICOS) scheme was utilized to construct the search strategy of this review. In the protocol we stated that the search strategy will search for concepts of intervention (prehabilitation), outcome (PND) and study design(RCT), and these concepts will be combined with the ‘AND’ operator. The final search strategy of this review additionally searched for concept of patient (aged), and combined this concept and other concepts with the ‘AND’ operator in order to improve the specificity of our search. |
| --- |
| Synthesis methods:  In the protocol we stated that a meta-analysis concerning the effect of cognitive prehabilitation programmes will be carried out if at least two studies used the homogeneous outcome measure. However, due to clinical and methodological heterogeneity between the trials, after discussion and consensus we decided that the available quantitative data were analysed narratively and presented through tabulation and description. In addition, we transparently reported, when available, the study outcomes of each trial through structured reporting of effects and calculated effect sizes with a 95% confidence intervals (CI) using RevMan 5 software. Additionally, we were not able to perform all predesigned analyses and subgroups analyses as described in the published protocol of this review and could not assess the impact of publication bias owing to the limited number of trials included. In future updates of this review, this will again be assessed. |
